# Supplementary material for: Person-centred transition programme to empower adolescents with congenital heart disease in the transition to adulthood: a study protocol for a hybrid randomised controlled trial (STEPSTONES project)
Source: BMJ Open. 2017 Apr 17;7(4):e014593. doi: 10.1136/bmjopen-2016-014593 (PMC5719650; doi:10.1136/bmjopen-2016-014593)
Supplement: Author's manuscript [file bmjopen-2016-014593.draft_revisions.pdf]

BMJ Open

A person-centered transition program to empower adolescents with congenital heart disease in the transition to adulthood: study protocol for a hybrid randomized controlled trial (STEPSTONES project)

Journal:	*BMJ Open*
Manuscript ID	bmjopen-2016-014593
Article Type:	Protocol
Date Submitted by the Author:	05-Oct-2016
Complete List of Authors:	Acuna Mora, Mariela; University of Gothenburg, Institute of Health and Care Sciences; KU Leuven, Department of Public Health and Primary Care<br>Sparud-Lundin, Carina; University of Gothenburg, Institute of Health and Care Sciences<br>Bratt, Ewa-Lena; University of Gothenburg, Institute of Health and Care Sciences; The Queen Silvia Children's Hospital, Department of Pediatric Cardiology<br>Moons, Philip; KU Leuven, Department of Public Health and Primary Care; University of Gothenborg, Institute of Health and Care Sciences
**Primary Subject Heading**:	Cardiovascular medicine
Secondary Subject Heading:	Research methods, Patient-centred medicine, Paediatrics
Keywords:	Congenital heart disease < CARDIOLOGY, Paediatric cardiology < PAEDIATRICS, Randomized controlled trial, Transitional care, Person-centered care

SCHOLARONE™
Manuscripts

A person-centered transition program to empower adolescents with congenital heart disease in the transition to adulthood: study protocol for a hybrid randomized controlled trial (STEPSTONES project)

Mariela Acuña Mora MSc, RN[1,2], Carina Sparud-Lundin PhD, RN[1], Ewa-Lena Bratt PhD, RN[1,3], Philip Moons PhD, RN[1,2]

[1] Institute of Health and Care Sciences, University of Gothenburg, Gothenburg, Sweden

[2] KU Leuven Department of Public Health and Primary Care, Leuven, Belgium

[3] Department of Pediatric Cardiology, The Queen Silvia Children's Hospital, Sweden

Mariela Acuña Mora: mariela.acuna.mora@gu.se

Carina Sparud-Lundin: carina.s-lundin@fhs.gu.se

Ewa-Lena Bratt: ewa-lena.bratt@gu.se

Philip Moons: philip.moons@med.kuleuven.be

Corresponding author:

Philip Moons, PhD, RN, Professor of Healthcare and Nursing Science, Department of Public Health and Primary Care, Kapucijnenvoer 35 Box7001 3000 Leuven-Belgium; Phone: +32 16 373315; philip.moons@kuleuven.be

Word Count: 5017

For peer review only - http://bmjopen.bmj.com/site/about/guidelines.xhtml

ABSTRACT

Introduction

When a young person grows up they evolve from an independent child to an empowered adult. If an individual has a chronic condition this additional burden may hamper an adequate development and independence. Transition programs for young persons with chronic disorders aim to provide the necessary skills for self-management and participation in care. However, strong evidence on the effects of these interventions is lacking. Therefore, as part of the STEPSTONES project (Swedish Transition Effects Project Supporting Teenagers with chrONic mEdical conditionS) we propose a trial to assess the effectiveness of a structured, person-centered transition program to empower adolescents with congenital heart disease in the transition to adulthood.

Methods/Design

STEPSTONES will use a hybrid experimental design in which a randomized controlled trial is embedded in a longitudinal, observational study. It will be conducted in four pediatric cardiology centers in Sweden. Two centers will be allocated to the randomized controlled trial group, assigning patients randomly to the intervention group (n=63) or the comparison group (n=63). The other two centers will form the intervention-naïve control group (n=63). The primary outcome is the level of patient empowerment, as measured by the Gothenburg Young Persons Empowerment Scale (GYPES).

Ethics and dissemination

The study has been approved by the Regional Ethical Board of Gothenburg, Sweden. Findings will be reported following the CONSORT statement and disseminated at international conferences and as published papers in peer-reviewed journals.

For peer review only

Trial registration number: NCT02675361

Keywords

Adolescents, congenital heart disease, heart defect, person centered care, randomized controlled trial, transfer, transition.

ARTICLE SUMMARY

Strengths and limitations

- This is a multicenter study that uses a novel study design to test the effectiveness of a person-centered transition program.
- Although several guidelines have recommended the implementation of transition interventions, the evidence of these is still insufficient. The study is expected to fill a gap in current transitional care knowledge.
- Due to the type of intervention, blinding of participants is not possible.
- A long recruitment period may require extending the trial and delaying data analysis.
- In order to have a long follow-up, transition interventions are recommended to start before the age of 16 years, which is not feasible in this study.

For peer review only - http://bmjopen.bmj.com/site/about/guidelines.xhtml

BACKGROUND

In developed countries, 10-40% of the adolescent population has a chronic disease with childhood onset.[1] As a result of improvements in diagnostic techniques and treatments, survival has increased, and 90% of them now reach adulthood.[2] Growing up with a chronic condition poses an extra burden on the individual, over and above the challenges typically related to adolescence. Management of the transition from childhood to adulthood and the shift from pediatric to adult care requires special attention from healthcare professionals. In healthcare, transition can be defined as the *"process by which adolescents and young adults with chronic childhood illnesses are prepared to take charge of their lives and their health in adulthood"*.[3]

To improve and facilitate this process, transition programs have been developed.[4] A well designed transition program is individually-tailored, flexible and age and developmentally appropriate.[5] The goal of transition programs is to support the person's adjustment to a new care culture, to provide the necessary tools for self-care and to help satisfy medical, psychosocial and educational needs.[6] Evidence on the effects of transition programs is scant, the level of existing evidence is low and the effectiveness of these interventions has yet to be determined.[7] This is due to the limited number of effectiveness studies; methodological issues (e.g. contamination of control groups has not been accounted for in prior studies) and a short follow-up period of 12 months maximum.[7]

Patient empowerment is an asset that relates to the adolescents' self-management and participation in care[8]. Empowered adolescents can interact better with the adult healthcare system, are more independent and more actively involved in decisions regarding their health.[9,10] However, patient empowerment has not been a formal target in transition programs so far. To achieve this, a person-centered care approach is an important.[11] This perspective allows for a partnership between the adolescent and healthcare providers in which the

For peer review only - http://bmjopen.bmj.com/site/about/guidelines.xhtml

adolescent's views guide the intervention.[12] Through this approach it is possible to share helpful resources and information with the adolescents and give them the opportunity to assume an active role in the decision-making process. [11]

Congenital heart disease (ConHD) is a typical example of a life-course disease and can therefore be considered a chronic condition. ConHD has a prevalence of 9.1 per 1000 newborns and represents 28% of all congenital defects.[13] Around 90% of children born with ConHD reach adulthood,[2] and approximately 6.2 in 1000 adults live with this condition.[14] Although several centers have established a transition program for individuals with ConHD,[15] only one study on the effectiveness of such a program has been undertaken so far. That study was conducted in Canada and used a quasi-experimental design to test the effects of a one-hour intervention led by a nurse.[16] Significant improvements in the level of knowledge and self-management among patients were observed.[16] In addition, a comparative and a longitudinal study in Belgium confirmed the effectiveness of patient education.[17, 18]

To address the gaps in evidence on the effectiveness of transition programs, we established the STEPSTONES project (Swedish Transition Effects Project Supporting Teenagers with chrONic mEdical conditionS). This project aims at developing and evaluating the effectiveness of transition programs for young persons with chronic childhood-onset conditions. Although the project has a generic nature, it will first study adolescents with ConHD.

Objective and hypothesis

This article describes the protocol for a study to test the effectiveness of a transition program to empower adolescents with congenital heart disease in the transition to adulthood and the transfer to adult care. The main hypothesis is that adolescents with ConHD who receive a

For peer review only - http://bmjopen.bmj.com/site/about/guidelines.xhtml
For peer review only

structured, person-centered transition program over a 2-year period have a higher patient empowerment score than adolescents receiving usual care.

This study is presented in accordance with the guidelines issued by SPIRIT for reporting of study protocols.[19]

For peer review only

METHODS/DESIGN

Study design and setting

A hybrid experimental design will be used, in which a randomized controlled trial (RCT) is embedded in a longitudinal, observational study. This will result in a 3-arm design (see Figure 1). The study will be conducted in four ConHD-centers in Sweden. Two of the centers are allocated to the RCT, where participants are randomly assigned to either the intervention group or the comparison group. The other two centers are designated as the control group and represent intervention-naïve centers.

This type of design allows for the investigation of the effectiveness of the transition program while also taking into account potential contamination of the comparison group in the intervention centers.

Sample size estimation

Sample size calculation is based on the primary outcome of patient empowerment. The target is an improved patient empowerment score of 5.25 points on a scale from 15-75 (i.e. 0.5 standard deviation). For two-sided tests with alpha=0.05 and power=80%, 63 patients are needed in each arm of the RCT. An additional 63 patients will be recruited in the centers of

For peer review only - http://bmjopen.bmj.com/site/about/guidelines.xhtml

the control group. Among the four centers a total of 189 patients will be enrolled (See Figure 1).

Participants and recruitment

Eligible participants are 1) adolescents with ConHD, defined as "*a gross structural abnormality of the heart or the intrathoracic great vessels that is actually or potentially of functional significance*";[20] 2) age 16 years; 3) Swedish speaking; and 4) literate.

Patients are excluded if they do not have the physical or cognitive capacity to complete the questionnaires, have acquired heart conditions or heart transplantation. Parents of the adolescents will also be asked to participate.

Randomization

Using a web-based system, a stratified block randomization with random variable block size will assign patients of the RCT centers to the intervention group or the comparison group (1:1).[21] Block randomization ensures that the transition coordinators (TCs) in the two centers have a relatively continuous exposure to the intervention over time, which allows them to keep their skills up-to-date and to ensure the fidelity of the intervention. The fact that the block sizes vary randomly minimizes the possibility that the TCs could predict group assignments.

Stratification is made by center and disease complexity. The stratification for center ensures that the two centers have an equal number of intervention patients. Stratification by disease complexity (mild; moderate; complex)[22] is important to avoid overrepresentation of

For peer review only - http://bmjopen.bmj.com/site/about/guidelines.xhtml

that people with, for example, complex ConHD, in one or the other group due to failing randomization.

Overall this approach decreases the within centers variability and reduces the risk for bias and confounding.[21]

Intervention

Transition program (intervention group)

This study implements a multicomponent intervention, adapted from a brief transition program developed by Hilderson.[23] Adaptation of the program followed the intervention mapping process.[24] Intervention mapping is a thorough method used to build comprehensive programs for health promotion and consists of 6 consecutive steps: 1) assessment of the needs of the target population; 2) defining program objectives by specifying performance and change objectives; 3) determining theory-based intervention methods and practical strategies; 4) designing and producing the intervention program; 5) adoption and implementation of the intervention; and 6) developing and executing a plan for evaluation.[24] Applying the intervention mapping method in this study ensures that the intervention is well-grounded and includes the necessary components to target the program objectives. A detailed description of each of the steps will be given in a related article.

The transition program includes 8 key components: a) a transition coordinator (TC); b) a written person-centered transition plan; c) provision of information and education about the condition, treatment and health behaviors; d) availability by telephone and email; e) information about and contact with the GUCH clinic (Grown-Up Congenital Heart Disease); f) guidance of parents; g) meeting with peers; and h) the actual transfer to the GUCH clinic.

For peer review only - http://bmjopen.bmj.com/site/about/guidelines.xhtml

These components are implemented in five steps: 1) a first patient visit with the TC; 2) second outpatient visit with TC; 3) information day for adolescents and their parents; 4) third outpatient visit with TC; and 5) actual transfer to the GUCH clinic. The TCs are specialized nurses at the outpatient clinic of pediatric cardiology that have received specific training in performing this intervention.

Throughout the intervention the TCs employ a person-centered care (PCC) approach. PCC implies that participants are active agents in their care and decision-making process.[12] Ekman proposes that PCC can be implemented in three stages.[12] First, initiating the partnership by allowing the patients to tell their story (patient narratives). This allows for the patients' views to be at the center of care. Second, developing the partnership through constant communication between the patient and healthcare providers. This is accomplished by sharing experiences and building a common understanding of the care plan. Third, safeguarding the partnership by documenting the decisions made and the preferences of the patients.

The content of the visits is individualized, age and developmentally adapted and it is documented in the individualized transfer plan. The TCs together with the adolescent are in charge of determining which topics are important to discuss, in establishing goals related to patient empowerment and in assessing the need for referral to other services.

Usual care (intervention, control and comparison group)

All adolescents who participate in the study receive usual care, irrespective of whether in the intervention, comparison or control group. Usual care includes visits to an outpatient pediatric cardiology clinic where they meet a nurse and a pediatric cardiologist. Disease

For peer review only

For peer review only - http://bmjopen.bmj.com/site/about/guidelines.xhtml

complexity determines the frequency of the visits. None of the participating centers have a TC as a part of usual care and there is no documentation of transition plans.

Since usual care can vary across clinics and over time, an assessment of transition-related practices will be done in all the centers at the beginning of the intervention and at the end of the study. This assessment will be comprised of three elements: 1) a structured survey called CHAT (Congenital Heart Adolescent and Transition) (on file); 2) interviews with healthcare professionals who are involved in the care for adolescents with ConHD in the participating centers; and 3) observations of the routine during the clinic visits.

Blinding

Due to the nature of the intervention and the study design, it is not possible to blind the participants, TCs or data collection officers (DCOs). However, the TCs and DCOs in charge of carrying out the intervention and data collection are not involved in the preparation of the intervention design or in the statistical analyses.

To avoid cross contamination between the comparison and the intervention group within the RCT centers, TCs are not involved in the delivery of care of the comparison group and other healthcare professionals in the participating centers will not be informed of the intervention components. DCOs in the control group do not participate in discussions of the intervention.

Intervention fidelity

For peer review only - http://bmjopen.bmj.com/site/about/guidelines.xhtml

For peer review only

To assure that the transition program is implemented according to the developed intervention, the TCs are selected based on their experience of working with children and adolescents with ConHD. In preparation for this study, they received a specifically designed 4-days training program, addressing adolescents' health and development; communicating with and interviewing young persons; patient education; person-centered care; and the detection of and screening for risk behaviors. Training is given by experts in the aforementioned fields and features different pedagogical approaches, e.g. group discussions, case assessments, and role play. In addition, the TCs were asked to read two textbooks on adolescent health and person-centered care.

Standard Operating Procedures (SOPs) for each group of the study were developed in order to ensure consistency throughout the study. These SOPs included: ethical approval; study preparation; study administration; participants recruitment and survey completion; intervention characteristics; usual care; data storage, entry and security; study progress; and publication policy.

Intervention fidelity is monitored as part of the process evaluation. The Medical Research Council (MRC) guidance on process evaluation of complex interventions is applied,[25] and both quantitative and qualitative methods will be used. In the quantitative assessment we investigate the acceptability, adherence/fidelity and attrition, as well as potential safety issues. These data are collected using three assessment forms (enrolment/follow-up form, adverse events report and intervention implementation form) filled out by the TCs during the intervention period. The qualitative assessment will obtain information about the experiences of and implementation by the TCs and will provide insights into the mechanisms of impact based on the experiences of patients and their parents. The process evaluation will be undertaken as a separate study conducted in parallel with this effectiveness study.

For peer review only - http://bmjopen.bmj.com/site/about/guidelines.xhtml

Follow-up period and data collection

During the 2-year follow-up period, outcome measurements are assessed on three different occasions: T0, baseline when the participants are 16 years old; T1, midterm when the participants are 17 years; and T2, final data collection when the participants have been transferred to adult care and are approximately 18.5-19 years old (See Figure 1).

Participants from the intervention group will be mailed the set of questionnaires one month before the outpatient clinic visit. If the questionnaires have not been received by the time of the visit, they will have the opportunity to complete them while waiting for their appointment. Patients from the control and comparison groups may not have a scheduled outpatient visit. Therefore, data collection in these groups is completely undertaken by post when the participants are 16 (T0), 17 (T1) and 18.5 years old (T2).

To minimize non-response rate in the comparison and control groups a modified Dillman procedure will be used.[26] Three weeks after the documents are sent, non-responders will receive a first personalized reminder. If after five weeks the questionnaires have not been received a second reminder letter and a new set of questionnaires is sent. After seven weeks, persistent non-responders are contacted by telephone and asked whether they received the questionnaires, if the mailing address is correct, if they want a new set of questionnaires and if they want to continue participating in the study. After 9 weeks a last reminder is sent for those who agree to participate in the study during the telephone call.

Outcome measurements

For peer review only - http://bmjopen.bmj.com/site/about/guidelines.xhtml

A background information questionnaire is included, comprising sociodemographic variables such as sex, age, educational level, size of community, place of birth, number of siblings, birth order, living situation, other health conditions and medication (obtained from self-report and medical file).

Standardized questionnaires are used to assess primary and secondary outcomes. Table 1 presents an overview of the characteristics and psychometric properties of the questionnaires used.

For peer review only

For peer review only - http://bmjopen.bmj.com/site/about/guidelines.xhtml

Table 1: Psychometric properties of the questionnaires used in STEPSTONES-ConHD

Variable	Subject	Measurements	Items	Validity	Reliability	Responsiveness	Interpretation
Primary Outcome							
Patient empowerment	AD	Gothenburg Young Persons Empowerment Scale (GYPES)	15	Construct validity confirmed in relation to the Empowerment theory. [9]<br><br>Face validity confirmed in young persons with chronic conditions (data on file)	Internal consistency confirmed in young persons with ConHD, α=0.821 (data on file)	Floor scores: 0%; Ceiling scores: 1.5% (data on file)	Score from 15-75. Higher score reflecting a higher level of patient empowerment
Secondary Outcomes							
Transition readiness	AD	Readiness for Transition Questionnaire (RTQ) adolescent version	26	Validity based on relationships with other variables confirmed in young persons with kidney transplant, [27] p.91-92	Internal consistency confirmed in young persons with kidney transplant; α values over 0.70,[27]	NR	Scores from 10-40. Higher scores denote increased adolescent or caregiver responsibility
	PA	Readiness for Transition Questionnaire (RTQ) parent version		Construct validity reported for parents of young persons with kidney transplant, [27] p.91-92	Reliability reported for parents of young persons with kidney transplant; α values over 0.70, [27] p.90	NR	
Knowledge on ConHD	AD	Knowledge Scale for Adults with Congenitally Malformed Hearts (KnoCoMH)	19	Validity was confirmed in adults with ConHD in relation to discrimination ability, the relationship of the items and the construct of interest, [28] p.232-234	Internal consistency and test-retest reliability confirmed,[28] p.232-234	NR	Scores are calculated by dichotomizing the answers (correct/incorrect) for each domain

For peer review only

Health behaviors	AD	Health Behavior Scale-Congenital Heart Disease (HBS-CHD)	15*	Item content validity, scale content validity index and validity based on relationships to other variables confirmed in adolescents with ConHD, [29] p.548	Stability not confirmed.[29]	Confirmed in adolescents with ConHD by Guyatt's Responsiveness Index, [29] p.551	Substance use, dental hygiene and total health risk score from 0-100. Physical exercise score from 0-∞
Patient-reported health		Pediatric Quality of Life Inventory 4.0 (PedsQL) generic module	23	Convergent validity, measurement invariance and factor structure confirmed in pediatric populations, [30] p.336-337 [31] p.1507-1510	Test-retest reliability, and internal consistency show α values over 0.70 in pediatric populations, [31] p.1507 [30] p.333-335	Minimal clinically important differences reported for young people with ConHD was 6.0 for the total score,[32]	Scores from 0 to 100. Higher scores indicate a better perceived health status
	AD	Pediatric Quality of Life Inventory 4.0 (PedsQL) cardiac module	27	Convergent validity was confirmed, [33] p.215	Internal consistency for majority of scales exceeded α values of 0.70, [33] p.214	Minimal clinically important differences reported for young people with ConHD ranged from 7.6 for symptoms to 12.6 for communication, [32]	Scores from 0 to 100. Higher scores indicate lower problems
		EQ-5D-3L	5	Convergent and discriminative validity confirmed in cardiovascular patients, [34] p.6	Test-retest reliability confirmed in cardiovascular patients, [34] p.6	Confirmed in cardiovascular patients, [34] p.6	Scores from 1 (no problems) to 3 (extreme problems) on five dimensions. Scores from 0 (worst imaginable health state) to 100

For peer review only

For peer review only - http://bmjopen.bmj.com/site/about/guidelines.xhtml

							(best imaginable health state)
Quality of life	AD	Linear analog scale (LAS)	1	Validity based on relationship with Satisfaction with Life Scale found to be highly correlated (ρ=0.52). Test content confirmed in adults with ConHD (100% of patients understood the wording and format), [35]	Test-retest reliability confirmed in adults with ConHD (ICC=0.65), [35]	Confirmed in adults with ConHD (Floor scores= 0%, ceiling scores=2.7%), [35]	Score is from 100 (best imaginable health status) to 0 (worst imaginable health status)
Healthcare utilization	AD	Healthcare utilization questionnaire	11	NA	NA	NA	NA
Parental uncertainty towards transfer to adult care	PA	Linear analog scale (LAS)	1	Face validity confirmed in parents of young persons with chronic conditions (data on file).<br>Test content confirmed in parents of young persons with ConHD (100% of patients understood the wording and format).	NR	Floor scores: 7.8%; Ceiling scores: 4.8% (data on file)	Score is from 0 (very uncertain about the transfer to adult care) to 100 (not uncertain about the transfer of care)
AD: adolescents. PA: parents. NA: not applicable. NR: not reported							

For peer review only

For peer review only - http://bmjopen.bmj.com/site/about/guidelines.xhtml

Primary outcome measures

The primary outcome is patient empowerment, defined as "*an enabling process or outcome arising from communication with the healthcare professional and a mutual sharing of resources over information relating to illness, which enhances the patient's feelings of control, self-efficacy, coping abilities and ability to achieve change over their condition.*[9] Empowerment includes five dimensions: 1) identity; 2) knowledge and understanding; 3) personal control; 4) decision making; and 5) enabling others.[9]

Since no questionnaire to measure the level of empowerment in young persons with chronic conditions exists, we developed the Gothenburg Young Persons Empowerment Scale-Congenital Heart Disease module (GYPES-ConHD). The scale has 15 items, 3 items for each of the aforementioned dimensions. Each item is answered on a five-point Likert scale: strongly disagree; disagree; neither agree or disagree; agree; and strongly agree. A total score is calculated by summing the item scores. The total score ranges from 15-75 points, with a higher score reflecting a higher level of patient empowerment.

Face validity was confirmed after cognitive interviews with 6 young persons with ConHD. The psychometric properties of this scale were examined in a cross-sectional study in 205 young persons with ConHD. Internal consistency of the items was confirmed with a Cronbach's alpha of 0.821. No floor or ceiling effect in scoring was observed. A complete description of the psychometric evaluation of GYPES will be provided in another article.

Secondary outcomes measures

We will measure six secondary outcomes in the adolescents: transition readiness, health behaviors, knowledge about ConHD, healthcare utilization, quality of life and patient-reported health. In the parents, we will measure transition readiness (proxy) and parental uncertainty toward transition.

For peer review only - http://bmjopen.bmj.com/site/about/guidelines.xhtml

Transition readiness is defined as the "*adolescent's readiness to assume complete responsibility for their healthcare and their readiness to transfer to adult medical care*".[27] Transition readiness is evaluated using the Readiness for Transition Questionnaire (RTQ), adolescent and parent versions. These questionnaires examine two aspects. First the overall transition readiness is assessed, using two items ranging from 1-4. The sum of these two items results in a score ranging from 2-8. Second, the frequency of adolescent responsibility and parental involvement are reported in ten different health behaviors, on a five-point Likert. Each variable results in a total score ranging from 10 to 40, higher scores indicate higher adolescent responsibility or parental involvement, respectively.

Adolescents' knowledge about their ConHD is assessed with the Knowledge Scale for adults with Congenital Malformed Hearts (KnoCoMH). [28] This instrument evaluates the level of knowledge within four domains: disease and its treatment; complications prevention; physical activity; and reproductive issues.[28] The questionnaire includes 19 items and scores are calculated by dichotomizing answers as correct or incorrect. Access to the medical files is needed in order to confirm the answers in some of the items. For participants where medical treatment or endocarditis prophylaxis is not recommended, the domains are excluded.

Health behaviors are measured with the Health Behavior Scale-CHD. Health behaviors are *"activities a person undertakes to maintain or improve health and prevent diseases"*.[29] This scale assesses alcohol consumption, tobacco use, dental care and physical activity. The scale has 15 items that help to calculate three summary risk scores: substance use (score 0-100), dental hygiene (score 0-100) and total health risk score (score 0-100). Higher risk scores represent unhealthier behaviors.

Patient-reported health is assessed by the generic and cardiac modules of the Pediatric Quality of Life inventory 4.0 (PedsQL 4.0), as well as the EuroQoL 5 dimensions 3 levels

For peer review only - http://bmjopen.bmj.com/site/about/guidelines.xhtml

(EQ-5D-3L). The PedsQL 4.0 provides information on health status in relation to psychosocial and physical health.[31] The generic module consists of 23 items that cover four dimensions: physical; emotional; social functioning; and school functioning. Items are answered by using a five-point Likert scale: never, almost never, sometimes, often, and always. For each dimension, a scale score can be calculated, which ranges from 0 to 100. Further, a physical health summary score, a psychosocial health summary score, and a total score can be calculated.

The cardiac module contains 27 items that cover six dimensions: symptoms; treatment; perceived physical appearance; treatment anxiety; cognitive problems; and communication. Each item is answered using a 5-point Likert scale (never, almost never, sometimes, often and always). As in the generic version, scores are transformed on a scale from 0 to 100, and then a mean subscale core is calculated.[33]

The EQ-5D-3L comprises a descriptive system and a visual analog scale (VAS). The descriptive system has 5 dimensions (mobility, self-care, usual activities, pain/discomfort and anxiety/depression) with 3 response categories per dimension.[36] The participants are asked to indicate which statement is most suitable in relation to their health status. The VAS measures the participants' self-rated health on a scale with the endpoints "best imaginable health possible" (=100) and "worst imaginable health state (=0)". The descriptive system allows for the determination of a 5 digit health state that combines the level of perceived problems in each dimension (level 1: indicating no problem, level 2: indicating some problems and level 3: indicating extreme problems). Information obtained from the descriptive system can be converted to a single summary index by assigning values to each level of every dimension.

Quality of life is defined as "*the overall degree of life satisfaction that is positively or negatively influenced by individuals' perception of certain aspects of life important to them,*

For peer review only

including matters both related and unrelated to health".[37] This variable is measured using a 10 cm linear analog scale (LAS),[35] where the participants indicate in the scale how they judge their current quality of life. The scale looks similar to the VAS of the EQ-5D, but has the endpoints "best imaginable quality of life" (=100) and "worst imaginable quality of life" (=0).

Healthcare utilization within the preceding six months is measured in relation to their ConHD and other possible conditions. The measurement consists of six dimensions: hospital stay; healthcare contact; specialized care; emergency care; school absence; and follow-up unit after transfer to adult care. This assessment was developed to be used in the APPROACH-IS project.[38]

Parental uncertainty is understood as the degree of certainty/uncertainty the parents have in relation to their children being transfer to adult care. This outcome is measured through LAS with the endpoints "extremely uncertain" (=100) and "not uncertain at all" (=0). This LAS has been developed for the purpose of the present study.

In addition to the primary and secondary endpoints, we will calculate a composite endpoint. We will do so because the transition program is a complex intervention that may have varying effects on different outcomes. Prior studies on the impact of transition programs showed that the intervention has impacted on transition readiness,[16] patient knowledge,[16] quality of life,[39] and psychosocial health.[39] Therefore, each patient will be categorized as improved or not. Patients will be classified as improved if they have an improvement in empowerment, transition readiness, knowledge, quality of life, or psychosocial health of 0.5 standard deviations compared to their baseline measurement.

Data management

For peer review only - http://bmjopen.bmj.com/site/about/guidelines.xhtml

Data are entered into a web-based system by the TC and the DCO. Those with access to the system are the TC, DCO, project coordinator and principal investigator, and each have a personal login. All data are checked for completeness and the quality of the data is monitored at least twice a year by the members of the research group. This process is done by verifying records with the raw data and checking frequency distributions to identify responses outside of the given possibilities.

Statistical analyses plan

The primary analyses will be done following the intention to treat principle which helps to avoid biases in the comparisons between groups. The primary endpoint (level of empowerment) will be analyzed using Fishers non-parametric permutation test unadjusted between the two groups. Complementary between-group analyses will be made using ANCOVA adjusted for baseline variables that differ significantly between groups and for each center.

All analyses will be predefined in a comprehensive statistical analysis plan before data lock. The level of significance will be at *p value* equal to or less than 0.05. Two-sided tests will be used.

Trial duration

Patient recruitment began July 2016 and is planned to finish in December 2017. Once recruited, patients are followed-up until they are transferred to GUCH. Data collection is expected to be finished by December 2019. After the data set is locked, analysis and dissemination of results will begin.

For peer review only - http://bmjopen.bmj.com/site/about/guidelines.xhtml

Ethical considerations

The study was approved by the Regional Ethical Board of Gothenburg, Sweden (No.931-15). Before inclusion in the study, we seek written informed consent from the adolescents and their parents after they have received written and oral information about the study. Participants have the right to withdraw at any point throughout the study. The principles established in the Declaration of Helsinki will be followed during the entire duration of the study.[40]

To ensure confidentiality personal data is coded. Participant and study-related information is stored in locked cabinets and digital documents are password-protected.

Follow-up on adverse events

All adverse events (AEs) must be reported to the principal investigator and project coordinator within a week. In case of a serious AE, the report must be made immediately. Appropriate measures will be undertaken in every AE in order to ensure patient safety.

Dissemination

The results of this study will be published in peer reviewed journals following the CONSORT statement.[41] Abstracts for poster or oral presentations will be submitted to international conferences. Authorship is granted to a collaborator from every participating center and members of the research group fulfilling the criteria of authorship [42, 43]. If possible, we will include "on behalf of STEPSTONES-ConHD" in the list of authors.

For peer review only - http://bmjopen.bmj.com/site/about/guidelines.xhtml

DISCUSSION

Previous studies have tried to determine the effectiveness of transition programs. However, due to several methodological limitations, the true effect of these interventions is still uncertain.[7] Therefore, we will conduct a study that assesses the effectiveness of a structured, person-centered transition program to empower adolescents with ConHD in the transition to adulthood. Through the implementation of the proposed transition program, we expect an increase in the level of empowerment. A high level of empowerment can help the adolescent navigate through the adult care system, participate in care planning, increase self-management and decision-making skills.[8]

The intervention in this trial was designed and initially evaluated by Hilderson,[23, 39] in Belgium. Therefore, it was important to determine if the intervention components were suitable in the Swedish setting. To accomplish this, five preparatory studies were done (four qualitative, one quantitative study). The qualitative studies included individual and focus groups interviews with young persons with ConHD and their parents.[44, 45] These studies provided an overview of the current transition practices in Sweden and the needs of this population before the transfer to adult care. The quantitative study was a cross-sectional study including 205 young persons with ConHD, and served as a pilot-test for our primary outcome measurement. Furthermore, the intervention was discussed with a panel of international experts with experience in transition research in adolescents with chronic conditions and patient representatives.

The preparatory studies and the discussion with the panel of international experts increased the quality, feasibility and acceptability of the intervention. This interdisciplinary

For peer review only - http://bmjopen.bmj.com/site/about/guidelines.xhtml

and comprehensive approach increases the chance of producing a high-quality intervention with positive results.[46]

The present study has several strengths, because we explicitly take limitations of prior studies into account. First, we employ a RCT which is the gold standard for the assessment of the effectiveness of interventions and provides the highest level of evidence. The stratified block randomization that we undertake ensures that the intervention and comparison groups are balanced and that potentially confounding factors, such as complexity of the disease or center characteristics, are equally distributed and therefore minimize the risk for bias the results.

Second, the proposed hybrid experimental design allows us to account for potential contamination of the control group. Indeed, the 3 arms allow performance of direct comparison between the intervention and comparison group while enabling a comparison of findings with those obtained in intervention-naive centers.

Third, patient empowerment is used as the primary outcome. Empowerment is argued to be important for the development and application of skills to promote behavioral changes in the adolescents.[10] Admittedly, empowerment can be seen both as a process and an outcome.[10] However, being an intermediate outcome, empowerment can be argued to be proximal to the intervention whereas continuing cardiac care, reduced morbidity or mortality are probably the ultimate, but more distal outcomes.

Fourth, the PCC approach that is used throughout the transition program is an important asset in the process of empowering patients.[11] Providing transitional care from a person-centered perspective is novel and has not been evaluated before in this age group.

Fifth, we follow-up the patients for more than 2 years. Doing so, we substantially expand the follow-up period of 12 months as used in prior studies.[7]

For peer review only - http://bmjopen.bmj.com/site/about/guidelines.xhtml

Sixth, since usual care may vary across centers and over time, we carry out a formal usual care assessment. Such a usual care assessment is important to draw firm conclusions and yet this is seldom performed in clinical trials.[47]

Irrespective of these strengths, we anticipate some limitations as well. First, we are not able to blind the participants or the healthcare team that will perform the intervention. To minimize a potential impact, neither the therapeutic team nor the TCs participate in the data analysis. Second, there is a possibility of a relatively long period of recruitment for participants which can require extending the trial period and delaying data analysis. Third, transition interventions have been advised to start early on, e.g. between 10-12 years of age, and have a long follow-up.[48] Although we recognize the importance of starting early, it would have jeopardized the feasibility of the study, because it would have taken almost a decade to finish recruitment and follow-up until the age of 18 years.

Conclusion

This article described the study protocol for a study to assess the effectiveness of a structured, person-centered transition program for young persons with ConHD. This study accounts for limitations of previous research and addresses an existing gap in the evidence of transitional care. The findings are expected to provide strong, high level evidence that proves the effects of this type of interventions in young persons with ConHD. If successful, this transition program can be implemented as part of routine care provided to young persons with ConHD, and transfer to other chronic conditions can be explored.

Trial status

For peer review only - http://bmjopen.bmj.com/site/about/guidelines.xhtml

Status of the trial at the time of submission: patient recruitment

List of abbreviations

ConHD: congenital heart disease

DCO: data collection officer

GYPES: Gothenburg Young Persons Empowerment Scale

GYPES-ConHD: Gothenburg Young Persons Empowerment Scale-Congenital Heart Disease module

GUCH: grown-up congenital heart disease unit

MRC: Medical Research Council

RCT: randomized controlled trial

STEPTONES: Swedish Transition Effects Project Supporting Teenagers with chrONic mEdical conditionS

TC: transition coordinator

Competing interests

There are no conflicts of interests.

Funding

For peer review only - http://bmjopen.bmj.com/site/about/guidelines.xhtml

This study is supported by research grants from the Swedish Heart-Lung Foundation; Swedish Research Council for Health, Working Life and Welfare; Swedish Children Heart Association; Swedish Research Council; and the Institute of Health and Care Sciences of the University of Gothenburg.

Author's contributions

MAM, CSL, ELB and PM conceived the study, were responsible for the design of the study protocol and drafted the manuscript. All authors critically revised and approved the manuscript.

Author's information

MAM is a PhD student at the Institute of Health and Care Sciences, University of Gothenburg, Sweden and at the Academic Center for Nursing and Midwifery, Department of Public Health and Primary Care, KU Leuven, Belgium. CSL is senior lecturer at the Institute of Health and Care Sciences, University of Gothenburg, Sweden. ELB is senior lecturer at the Institute of Health and Care Sciences, University of Gothenburg, Sweden and a pediatric nurse in Queen Silvia Children's Hospital. PM is Professor of Healthcare and Nursing Science and Program Director at the KU Leuven Department of Public Health and Primary Care, Belgium and Visiting Professor at the Institute of Health and Care Sciences, University of Gothenburg, Sweden.

Acknowledgements

For peer review only - http://bmjopen.bmj.com/site/about/guidelines.xhtml

We would like to acknowledge the collaboration from the members of the panel of international experts and of the Advisory Board.

References

1. Kennedy A, Sloman F, Douglass JA, et al. Young people with chronic illness: the approach to transition. Intern Med J 2007;**37**(8):555-60.

2. Moons P, Bovijn L, Budts W, et al. Temporal trends in survival to adulthood among patients born with congenital heart disease from 1970 to 1992 in Belgium. Circulation 2010;**122**(22):2264-72.

3. Meadows AK, Bosco V, Tong E, et al. Transition and transfer from pediatric to adult care of young adults with complex congenital heart disease. Curr Cardiol Rep 2009;**11**(4):291-97.

4. American Academy of Pediatrics. A consensus statement on health care transitions for young adults with special health care needs. Pediatrics 2002;**110**(6 Pt 2):1304-6.

5. Warnes CA, Williams RG, Bashore TM, et al. ACC/AHA 2008 Guidelines for the Management of Adults With Congenital Heart Disease: A Report of the American College of Cardiology/American Heart Association Task Force on Practice Guidelines (Writing Committee to Develop Guidelines on the Management of Adults With Congenital Heart Disease): Developed in Collaboration With the American Society of Echocardiography, Heart Rhythm Society, International Society for Adult Congenital Heart Disease, Society for Cardiovascular Angiography and Interventions, and Society of Thoracic Surgeons. Circulation 2008;**118**(23):E714-E833.

6. Crowley R, Wolfe I, Lock K, et al. Improving the transition between paediatric and adult healthcare: a systematic review. Arch Dis Child 2011;**96**(6):548-53.

7. Campbell F, Biggs K, Aldiss SK, et al. Transition of care for adolescents from paediatric services to adult health services. Cochrane Database Syst Rev 2016;**4**:CD009794.

8. Bravo P, Edwards A, Barr PJ, et al. Conceptualising patient empowerment: a mixed methods study. BMC Health Serv Res 2015;**15**:252.

For peer review only - http://bmjopen.bmj.com/site/about/guidelines.xhtml

9. Small N, Bower P, Chew-Graham CA, et al. Patient empowerment in long-term conditions: development and preliminary testing of a new measure. BMC Health Serv Res 2013;**13**:263.

10. Anderson RM, Funnell MM. Patient empowerment: Myths and misconceptions. Patient Educ Couns 2010;**79**(3):277-82.

11. Holmstrom I, Roing M. The relation between patient-centeredness and patient empowerment: a discussion on concepts. Patient Educ Couns 2010;**79**(2):167-72.

12. Ekman I, Swedberg K, Taft C, et al. Person-centered care--ready for prime time. Eur J Cardiovasc Nurs 2011;**10**(4):248-51.

13. Dolk H, Loane M, Garne E, et al. Congenital heart defects in Europe: prevalence and perinatal mortality, 2000 to 2005. Circulation 2011;**123**(8):841-9.

14. Marelli AJ, Ionescu-Ittu R, Mackie AS, et al. Lifetime prevalence of congenital heart disease in the general population from 2000 to 2010. Circulation 2014;**130**(9):749-56.

15. Hilderson D, Saidi AS, Van Deyk K, et al. Attitude toward and current practice of transfer and transition of adolescents with congenital heart disease in the United States of America and Europe. Pediatr Cardiol 2009;**30**(6):786-93.

16. Mackie AS, Islam S, Magill-Evans J, et al. Healthcare transition for youth with heart disease: a clinical trial. Heart 2014;**100**(14):1113-8.

17. Goossens E, Fieuws S, Van Deyk K, et al. Effectiveness of structured education on knowledge and health behaviors in patients with congenital heart disease. J Pediatr 2015;**166**(6):1370-6 e1.

18. Goossens E, Van Deyk K, Zupancic N, et al. Effectiveness of structured patient education on the knowledge level of adolescents and adults with congenital heart disease. Eur J Cardiovasc Nurs 2014;**13**(1):63-70.

19. Chan AW, Tetzlaff JM, Altman DG, et al. SPIRIT 2013 statement: defining standard protocol items for clinical trials. Ann Intern Med 2013;**158**(3):200-7.

20. Mitchell SC, Korones SB, Berendes HW. Congenital heart disease in 56,109 births. Incidence and natural history. Circulation 1971;**43**(3):323-32.

21. Efird J. Blocked randomization with randomly selected block sizes. Int J Environ Res Public Health 2011;**8**(1):15-20.

For peer review only - http://bmjopen.bmj.com/site/about/guidelines.xhtml

22. Warnes CA, Liberthson R, Danielson GK, et al. Task force 1: the changing profile of congenital heart disease in adult life. J Am Coll Cardiol 2001;**37**(5):1170-5.

23. Hilderson D, Westhovens R, Wouters C, et al. Rationale, design and baseline data of a mixed methods study examining the clinical impact of a brief transition programme for young people with juvenile idiopathic arthritis: the DON'T RETARD project. BMJ Open 2013;**3**(12):e003591.

24. Bartholomew LK, Markham CM, Ruiter R, et al. *Planning health promotion programs : an intervention mapping approach*. 4th ed. San Francisco: Jossey-Bass, 2016.

25. Moore GF, Audrey S, Barker M, et al. Process evaluation of complex interventions: Medical Research Council guidance. BMJ 2015;**350**:h1258.

26. Dillman D. Mail and other self-administered questionnaires. In: Rossi PH, Wright JD, Anderson AB, eds. Handbook of survey research. New York: Academic Press, 1983:pp. 359–77.

27. Gilleland J, Amaral S, Mee L, et al. Getting ready to leave: transition readiness in adolescent kidney transplant recipients. J Pediatr Psychol 2012;**37**(1):85-96.

28. Ronning H, Arestedt K, Erik Nielsen N, et al. Development and psychometric evaluation of the knowledge scale for adults with congenitally malformed hearts. J Cardiovasc Nurs 2013;**28**(3):228-37.

29. Goossens E, Luyckx K, Mommen N, et al. Health risk behaviors in adolescents and emerging adults with congenital heart disease: psychometric properties of the Health Behavior Scale-Congenital Heart Disease. Eur J Cardiovasc Nurs 2013;**12**(6):544-57.

30. Varni JW, Burwinkle TM, Seid M, et al. The PedsQL™* 4.0 as a Pediatric Population Health Measure: Feasibility, Reliability, and Validity. Ambul Pediatr 2003;**3**(6):329-41.

31. Petersen S, Hagglof B, Stenlund H, et al. Psychometric properties of the Swedish PedsQL, Pediatric Quality of Life Inventory 4.0 generic core scales. Acta Paediatr 2009;**98**(9):1504-12.

32. Bratt E-L, Luyckx K, Goossens E, et al. Patient-Reported Health in Young People With Congenital Heart Disease Transitioning to Adulthood. J Adolesc Health 2015;**57**(6):658-65.

33. Sand P, Kljajić M, Sunnegårdh J. The reliability of the Pediatric Quality of Life Inventory 3.0 Cardiac Module™ for Swedish children with congenital heart defects. Nordic Psychology 2013;**65**(3):210-23.

For peer review only - http://bmjopen.bmj.com/site/about/guidelines.xhtml

For peer review only

34. Dyer MT, Goldsmith KA, Sharples LS, et al. A review of health utilities using the EQ-5D in studies of cardiovascular disease. Health and Quality of Life Outcomes 2010;**8**(1):1-12.

35. Moons P, Van Deyk K, De Bleser L, et al. Quality of life and health status in adults with congenital heart disease: a direct comparison with healthy counterparts. Eur J Cardiovasc Prev Rehabil 2006;**13**(3):407-13.

36. EuroQoL Group. EuroQol--a new facility for the measurement of health-related quality of life. Health Policy 1990;**16**(3):199-208.

37. Moons P, Marquet K, Budts W, et al. Validity, reliability and responsiveness of the "Schedule for the Evaluation of Individual Quality of Life – Direct Weighting" (SEIQoL-DW) in congenital heart disease. Health and Quality of Life Outcomes 2004;**2**(1):1-8.

38. Apers S, Kovacs AH, Luyckx K, et al. Assessment of Patterns of Patient-Reported Outcomes in Adults with Congenital Heart disease - International Study (APPROACH-IS): rationale, design, and methods. Int J Cardiol 2015;**179**:334-42.

39. Hilderson D, Moons P, Van der Elst K, et al. The clinical impact of a brief transition programme for young people with juvenile idiopathic arthritis: results of the DON'T RETARD project. Rheumatology (Oxford) 2016;**55**(1):133-42.

40. World Medical Association. World medical association declaration of helsinki: Ethical principles for medical research involving human subjects. JAMA 2013;**310**(20):2191-94.

41. Moher D, Hopewell S, Schulz KF, et al. CONSORT 2010 explanation and elaboration: updated guidelines for reporting parallel group randomised trials. BMJ 2010;**340**:c869.

42. Bourbonniere MC, Russell DJ, Goldsmith CH. Authorship issues: one research center's experience with developing author guidelines. Am J Occup Ther 2006;**60**(1):111-7.

43. Whellan DJ, Ellis SJ, Kraus WE, et al. Method for establishing authorship in a multicenter clinical trial. Ann Intern Med 2009;**151**(6):414-20.

44. Asp A, Bratt EL, Bramhagen AC. Transfer to Adult Care--Experiences of Young Adults with Congenital Heart Disease. J Pediatr Nurs 2015;**30**(5):e3-10.

45. Burstrom A, Ojmyr-Joelsson M, Bratt EL, et al. Adolescents With Congenital Heart Disease and Their Parents: Needs Before Transfer to Adult Care. J Cardiovasc Nurs 2015.

For peer review only - http://bmjopen.bmj.com/site/about/guidelines.xhtml

46. Craig P, Dieppe P, Macintyre S, et al. Developing and evaluating complex interventions: the new Medical Research Council guidance. BMJ : British Medical Journal 2008;**337**:a1655.

47. Erlen JA, Tamres LK, Reynolds N, et al. Assessing usual care in clinical trials. West J Nurs Res 2015;**37**(3):288-98.

48. Sable C, Foster E, Uzark K, et al. Best practices in managing transition to adulthood for adolescents with congenital heart disease: the transition process and medical and psychosocial issues: a scientific statement from the American Heart Association. Circulation 2011;**123**(13):1454-85

For peer review only

For peer review only - http://bmjopen.bmj.com/site/about/guidelines.xhtml

Figure 1: Overview of study design and implementation steps

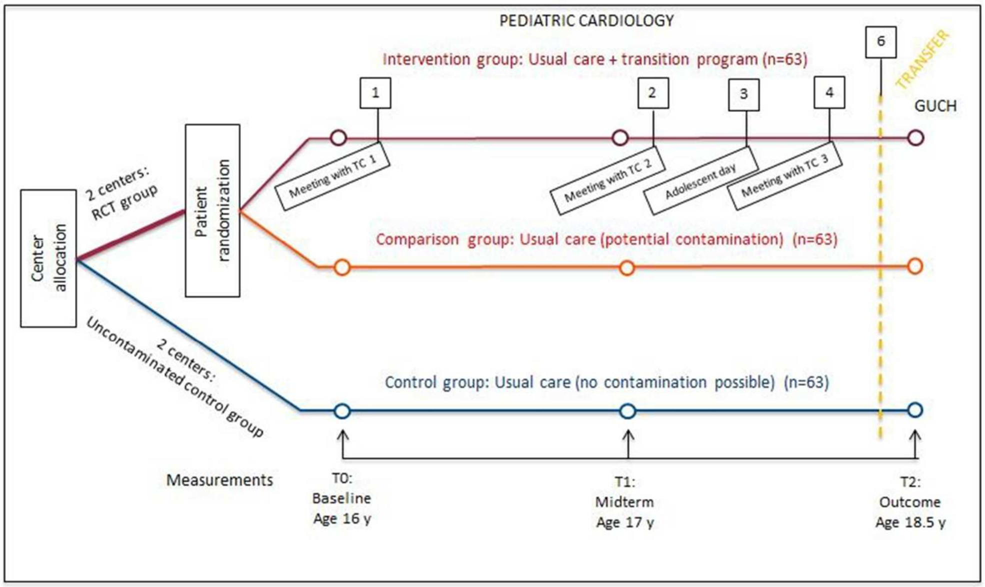

RCT: randomized controlled trial; TC: transition coordinator; GUCH: grown-up congenital heart disease clinic

Figure 1: Overview of study design and implementation steps

210x148mm (96 x 96 DPI)

STANDARD PROTOCOL ITEMS: RECOMMENDATIONS FOR INTERVENTIONAL TRIALS

SPIRIT 2013 Checklist: Recommended items to address in a clinical trial protocol and related documents*

Section/item	Item No	Description	Addressed on page number
**Administrative information**			
Title	1	Descriptive title identifying the study design, population, interventions, and, if applicable, trial acronym	1
Trial registration	2a	Trial identifier and registry name. If not yet registered, name of intended registry	3
	2b	All items from the World Health Organization Trial Registration Data Set	NA
Protocol version	3	Date and version identifier	NA
Funding	4	Sources and types of financial, material, and other support	27
Roles and responsibilities	5a	Names, affiliations, and roles of protocol contributors	27
	5b	Name and contact information for the trial sponsor	NA
	5c	Role of study sponsor and funders, if any, in study design; collection, management, analysis, and interpretation of data; writing of the report; and the decision to submit the report for publication, including whether they will have ultimate authority over any of these activities	NA
	5d	Composition, roles, and responsibilities of the coordinating centre, steering committee, endpoint adjudication committee, data management team, and other individuals or groups overseeing the trial, if applicable (see Item 21a for data monitoring committee)	NA

For peer review only

**Introduction**			
Background and rationale	6a	Description of research question and justification for undertaking the trial, including summary of relevant studies (published and unpublished) examining benefits and harms for each intervention	4-5
	6b	Explanation for choice of comparators	4-5
Objectives	7	Specific objectives or hypotheses	5-6
Trial design	8	Description of trial design including type of trial (eg, parallel group, crossover, factorial, single group), allocation ratio, and framework (eg, superiority, equivalence, noninferiority, exploratory)	6
**Methods: Participants, interventions, and outcomes**			
Study setting	9	Description of study settings (eg, community clinic, academic hospital) and list of countries where data will be collected. Reference to where list of study sites can be obtained	6
Eligibility criteria	10	Inclusion and exclusion criteria for participants. If applicable, eligibility criteria for study centres and individuals who will perform the interventions (eg, surgeons, psychotherapists)	7
Interventions	11a	Interventions for each group with sufficient detail to allow replication, including how and when they will be administered	8-10
	11b	Criteria for discontinuing or modifying allocated interventions for a given trial participant (eg, drug dose change in response to harms, participant request, or improving/worsening disease)	NA
	11c	Strategies to improve adherence to intervention protocols, and any procedures for monitoring adherence (eg, drug tablet return, laboratory tests)	11
	11d	Relevant concomitant care and interventions that are permitted or prohibited during the trial	9-10
Outcomes	12	Primary, secondary, and other outcomes, including the specific measurement variable (eg, systolic blood pressure), analysis metric (eg, change from baseline, final value, time to event), method of aggregation (eg, median, proportion), and time point for each outcome. Explanation of the clinical relevance of chosen efficacy and harm outcomes is strongly recommended	12-20
Participant timeline	13	Time schedule of enrolment, interventions (including any run-ins and washouts), assessments, and visits for participants. A schematic diagram is highly recommended (see Figure)	12, (see Figure 1)

For peer review only - http://bmjopen.bmj.com/site/about/guidelines.xhtml

Sample size	14	Estimated number of participants needed to achieve study objectives and how it was determined, including clinical and statistical assumptions supporting any sample size calculations	6-7
Recruitment	15	Strategies for achieving adequate participant enrolment to reach target sample size	NR

Methods: Assignment of interventions (for controlled trials)

Allocation:			
Sequence generation	16a	Method of generating the allocation sequence (eg, computer-generated random numbers), and list of any factors for stratification. To reduce predictability of a random sequence, details of any planned restriction (eg, blocking) should be provided in a separate document that is unavailable to those who enrol participants or assign interventions	7
Allocation concealment mechanism	16b	Mechanism of implementing the allocation sequence (eg, central telephone; sequentially numbered, opaque, sealed envelopes), describing any steps to conceal the sequence until interventions are assigned	7
Implementation	16c	Who will generate the allocation sequence, who will enrol participants, and who will assign participants to interventions	7
Blinding (masking)	17a	Who will be blinded after assignment to interventions (eg, trial participants, care providers, outcome assessors, data analysts), and how	10
	17b	If blinded, circumstances under which unblinding is permissible, and procedure for revealing a participant's allocated intervention during the trial	NA

Methods: Data collection, management, and analysis

Data collection methods	18a	Plans for assessment and collection of outcome, baseline, and other trial data, including any related processes to promote data quality (eg, duplicate measurements, training of assessors) and a description of study instruments (eg, questionnaires, laboratory tests) along with their reliability and validity, if known. Reference to where data collection forms can be found, if not in the protocol	12, 14-16
	18b	Plans to promote participant retention and complete follow-up, including list of any outcome data to be collected for participants who discontinue or deviate from intervention protocols	12

For peer review only - http://bmjopen.bmj.com/site/about/guidelines.xhtml

Data management	19	Plans for data entry, coding, security, and storage, including any related processes to promote data quality (eg, double data entry; range checks for data values). Reference to where details of data management procedures can be found, if not in the protocol	21
Statistical methods	20a	Statistical methods for analysing primary and secondary outcomes. Reference to where other details of the statistical analysis plan can be found, if not in the protocol	21
	20b	Methods for any additional analyses (eg, subgroup and adjusted analyses)	21
	20c	Definition of analysis population relating to protocol non-adherence (eg, as randomised analysis), and any statistical methods to handle missing data (eg, multiple imputation)	NR
**Methods: Monitoring**			
Data monitoring	21a	Composition of data monitoring committee (DMC); summary of its role and reporting structure; statement of whether it is independent from the sponsor and competing interests; and reference to where further details about its charter can be found, if not in the protocol. Alternatively, an explanation of why a DMC is not needed	NA
	21b	Description of any interim analyses and stopping guidelines, including who will have access to these interim results and make the final decision to terminate the trial	NA
Harms	22	Plans for collecting, assessing, reporting, and managing solicited and spontaneously reported adverse events and other unintended effects of trial interventions or trial conduct	22
Auditing	23	Frequency and procedures for auditing trial conduct, if any, and whether the process will be independent from investigators and the sponsor	NA
**Ethics and dissemination**			
Research ethics approval	24	Plans for seeking research ethics committee/institutional review board (REC/IRB) approval	21-22
Protocol amendments	25	Plans for communicating important protocol modifications (eg, changes to eligibility criteria, outcomes, analyses) to relevant parties (eg, investigators, REC/IRBs, trial participants, trial registries, journals, regulators)	NA

For peer review only - http://bmjopen.bmj.com/site/about/guidelines.xhtml

Consent or assent	26a	Who will obtain informed consent or assent from potential trial participants or authorised surrogates, and how (see Item 32)	22
	26b	Additional consent provisions for collection and use of participant data and biological specimens in ancillary studies, if applicable	NA
Confidentiality	27	How personal information about potential and enrolled participants will be collected, shared, and maintained in order to protect confidentiality before, during, and after the trial	22
Declaration of interests	28	Financial and other competing interests for principal investigators for the overall trial and each study site	26
Access to data	29	Statement of who will have access to the final trial dataset, and disclosure of contractual agreements that limit such access for investigators	NR
Ancillary and post-trial care	30	Provisions, if any, for ancillary and post-trial care, and for compensation to those who suffer harm from trial participation	NA
Dissemination policy	31a	Plans for investigators and sponsor to communicate trial results to participants, healthcare professionals, the public, and other relevant groups (eg, via publication, reporting in results databases, or other data sharing arrangements), including any publication restrictions	22
	31b	Authorship eligibility guidelines and any intended use of professional writers	22
	31c	Plans, if any, for granting public access to the full protocol, participant-level dataset, and statistical code	NA
**Appendices**			
Informed consent materials	32	Model consent form and other related documentation given to participants and authorised surrogates	Available upon request
Biological specimens	33	Plans for collection, laboratory evaluation, and storage of biological specimens for genetic or molecular analysis in the current trial and for future use in ancillary studies, if applicable	NA

*It is strongly recommended that this checklist be read in conjunction with the SPIRIT 2013 Explanation & Elaboration for important clarification on the items. Amendments to the protocol should be tracked and dated. The SPIRIT checklist is copyrighted by the SPIRIT Group under the Creative Commons "Attribution-NonCommercial-NoDerivs 3.0 Unported" license.

For peer review only - http://bmjopen.bmj.com/site/about/guidelines.xhtml

BMJ Open

A person-centered transition program to empower adolescents with congenital heart disease in the transition to adulthood: study protocol for a hybrid randomized controlled trial (STEPSTONES project)

Journal:	*BMJ Open*
Manuscript ID	bmjopen-2016-014593.R1
Article Type:	Protocol
Date Submitted by the Author:	12-Dec-2016

Complete List of Authors:	Acuna Mora, Mariela; University of Gothenburg, Institute of Health and Care Sciences; KU Leuven, Department of Public Health and Primary Care<br>Sparud-Lundin, Carina; University of Gothenburg, Institute of Health and Care Sciences<br>Bratt, Ewa-Lena; University of Gothenburg, Institute of Health and Care Sciences; The Queen Silvia Children's Hospital, Department of Pediatric Cardiology<br>Moons, Philip; KU Leuven, Department of Public Health and Primary Care; University of Gothenborg, Institute of Health and Care Sciences

**Primary Subject Heading**:	Cardiovascular medicine
Secondary Subject Heading:	Research methods, Patient-centred medicine, Paediatrics
Keywords:	Congenital heart disease < CARDIOLOGY, Paediatric cardiology < PAEDIATRICS, Randomized controlled trial, Transitional care, Person-centered care

SCHOLARONE™
Manuscripts

For peer review only

A person-centered transition program to empower adolescents with congenital heart disease in the transition to adulthood: study protocol for a hybrid randomized controlled trial (STEPSTONES project)

Mariela Acuña Mora MSc, RN[1,2], *Carina Sparud-Lundin PhD, RN*[1], *Ewa-Lena Bratt PhD, RN*[1,3], *Philip Moons PhD, RN*[1,2]

[1] Institute of Health and Care Sciences, University of Gothenburg, Gothenburg, Sweden

[2] KU Leuven Department of Public Health and Primary Care, Leuven, Belgium

[3] Department of Pediatric Cardiology, The Queen Silvia Children's Hospital, Sweden

Mariela Acuña Mora: mariela.acuna.mora@gu.se

Carina Sparud-Lundin: carina.s-lundin@fhs.gu.se

Ewa-Lena Bratt: ewa-lena.bratt@gu.se

Philip Moons: philip.moons@kuleuven.be

Corresponding author:

Philip Moons, PhD, RN, Professor of Healthcare and Nursing Science, Department of Public Health and Primary Care, Kapucijnenvoer 35 Box7001 3000 Leuven-Belgium; Phone: +32 16 373315; philip.moons@kuleuven.be

Word Count: 5241

For peer review only

ABSTRACT

Introduction

When a young person grows up they evolve from an independent child to an empowered adult. If an individual has a chronic condition this additional burden may hamper adequate development and independence. Transition programs for young persons with chronic disorders aim to provide the necessary skills for self-management and participation in care. However, strong evidence on the effects of these interventions is lacking. Therefore, as part of the STEPSTONES project (Swedish Transition Effects Project Supporting Teenagers with chrONic mEdical conditionS) we propose a trial to assess the effectiveness of a structured, person-centered transition program to empower adolescents with congenital heart disease in the transition to adulthood.

Methods/Design

STEPSTONES will use a hybrid experimental design in which a randomized controlled trial is embedded in a longitudinal, observational study. It will be conducted in four pediatric cardiology centers in Sweden. Two centers will be allocated to the randomized controlled trial group, assigning patients randomly to the intervention group (n=63) or the comparison group (n=63). The other two centers will form the intervention-naïve control group (n=63). The primary outcome is the level of patient empowerment, as measured by the Gothenburg Young Persons Empowerment Scale (GYPES).

Ethics and dissemination

The study has been approved by the Regional Ethical Board of Gothenburg, Sweden. Findings will be reported following the CONSORT statement and disseminated at international conferences and as published papers in peer-reviewed journals.

For peer review only

Trial registration number: NCT02675361

Keywords

Adolescents, congenital heart disease, heart defect, person centered care, randomized controlled trial, transfer, transition.

ARTICLE SUMMARY

Strengths and limitations

- This is a multicenter study that uses a novel study design to test the effectiveness of a person-centered transition program.
- Although several guidelines have recommended the implementation of transition interventions, the evidence of these is still insufficient. The study is expected to fill a gap in current transitional care knowledge.
- Due to the type of intervention, blinding of participants is not possible.
- In order to have a long follow-up, transition interventions are recommended to start before the age of 16 years, which is not feasible in this study.

For peer review only

For peer review only - http://bmjopen.bmj.com/site/about/guidelines.xhtml

BACKGROUND

In developed countries, 10-40% of the adolescent population has a chronic disease with childhood onset.[1] As a result of improvements in diagnostic techniques and treatments, survival has increased, and 90% of them now reach adulthood.[2] Growing up with a chronic condition poses an extra burden on the individual, over and above the challenges typically related to adolescence. Management of the transition from childhood to adulthood and the shift from pediatric to adult care requires special attention from healthcare professionals. The movement to adult care is known as transfer, defined as "*an event or series of events through which adolescents and young adults with chronic physical and medical conditions move their care from a pediatric to an adult health care environment*".[3] This transfer should be preceded by a preparatory transitional phase. In healthcare, transition can be defined as the *"process by which adolescents and young adults with chronic childhood illnesses are prepared to take charge of their lives and their health in adulthood"*.[3] To improve and facilitate this process, transition programs have been developed.[4] A well designed transition program is individually-tailored, flexible and age and developmentally appropriate.[5] The goal of transition programs is to support the person's adjustment to a new care culture, to provide the necessary tools for self-care and to help satisfy medical, psychosocial and educational needs.[6] Evidence on the effects of transition programs is scant, the level of existing evidence is low and the effectiveness of these interventions has yet to be determined.[7] This is due to the limited number of effectiveness studies; methodological issues (e.g. contamination of control groups has not been accounted for in prior studies) and a short follow-up period of 12 months maximum.[7]

Patient empowerment is an asset that relates to the adolescents' self-management and participation in care[8]. Empowered adolescents can interact better with the adult healthcare system, are more independent and more actively involved in decisions regarding their

For peer review only - http://bmjopen.bmj.com/site/about/guidelines.xhtml

health.[9,10] However, patient empowerment has not been a formal target in transition programs so far. To increase the level of empowerment, a person-centered care approach is important.[11] This perspective allows for a partnership between the adolescent and healthcare providers in which the adolescent's views guide the intervention.[12] Through this approach it is possible to share helpful resources and information with the adolescents and give them the opportunity to assume an active role in the decision-making process. [11]

Congenital heart disease (CHD) is a typical example of a life-course disease and can therefore be considered a chronic condition. CHD has a prevalence of 9.1 per 1000 newborns and represents 28% of all congenital defects.[13] Around 90% of children born with CHD reach adulthood,[2] and approximately 6.2 in 1000 adults live with this condition.[14] Although several centers have established a transition program for individuals with CHD,[15] only one study on the effectiveness of such a program has been undertaken so far. That study was conducted in Canada and used a quasi-experimental design to test the effects of a one-hour intervention led by a nurse.[16] Significant improvements in the level of knowledge and self-management among patients were observed.[16] In addition, a comparative and a longitudinal study in Belgium confirmed the effectiveness of patient education.[17, 18]

To address the gaps in evidence on the effectiveness of transition programs, we established the STEPSTONES project (Swedish Transition Effects Project Supporting Teenagers with chrONic mEdical conditionS). This project aims at developing and evaluating the effectiveness of transition programs for young persons with chronic childhood-onset conditions. Although the project will encompass different chronic conditions, it will first study adolescents with CHD.

Objective and hypothesis

For peer review only - http://bmjopen.bmj.com/site/about/guidelines.xhtml

This article describes the protocol for a study to test the effectiveness of a transition program to empower adolescents with congenital heart disease in the transition to adulthood and the transfer to adult care. The main hypothesis is that adolescents with CHD who receive a structured, person-centered transition program over a 2-year period have a higher patient empowerment score than adolescents receiving usual care.

This study is presented in accordance with the guidelines issued by SPIRIT for reporting of study protocols.[19]

For peer review only

METHODS/DESIGN

Study design and setting

A hybrid experimental design will be used, in which a randomized controlled trial (RCT) is embedded in a longitudinal, observational study. This will result in a 3-arm design (see Figure 1). The study will be conducted in four CHD-centers in Sweden. Two of the centers are allocated to the RCT, where participants are randomly assigned to either the intervention group or the comparison group. The other two centers are designated as the control group and represent intervention-naïve centers.

This type of design allows for the investigation of the effectiveness of the transition program while also taking into account potential contamination of the comparison group in the intervention centers.

Sample size estimation

For peer review only - http://bmjopen.bmj.com/site/about/guidelines.xhtml

Sample size calculation is based on the primary outcome of patient empowerment. The target is an improved patient empowerment score of 5.25 points on a scale from 15-75 (i.e. 0.5 standard deviation). For two-sided tests with alpha=0.05 and power=80%, 63 patients are needed in each arm of the RCT. In order to compensate for a potential 10% dropout rate, we will recruit 70 patients in each arm of the RCT. An additional 70 patients will be recruited in the centers of the control group. Among the four centers a total of 210 patients will be enrolled (See Figure 1).

Participants and recruitment

Eligible participants are 1) adolescents with CHD, defined as "*a gross structural abnormality of the heart or the intrathoracic great vessels that is actually or potentially of functional significance*";[20] 2) age 16 years; 3) Swedish speaking; and 4) literate.

Patients are excluded if they do not have the physical or cognitive capacity to complete the questionnaires, have acquired heart conditions or heart transplantation. Parents of the adolescents will also be asked to participate. All eligible participants are recruited by a transition coordinator (TC) or a data collection officer (DCO).

Randomization

Using a web-based system, a stratified block randomization with random variable block size will assign patients of the RCT centers to the intervention group or the comparison group (1:1).[21] Block randomization ensures that the transition coordinators (TCs) in the two centers have a relatively continuous exposure to the intervention over time, which allows them to keep their skills up-to-date and to ensure the fidelity of the intervention. The fact that the

For peer review only - http://bmjopen.bmj.com/site/about/guidelines.xhtml

block sizes vary randomly minimizes the possibility that the TCs could predict group assignments.

Stratification is made by center and disease complexity. The stratification for center ensures that the two centers have an equal number of intervention patients. Stratification by disease complexity (mild; moderate; complex)[22] is important to avoid overrepresentation of that people with, for example, complex CHD, in one or the other group due to failing randomization.

Overall this approach decreases the within centers variability and reduces the risk for bias and confounding.[21] The TCs are responsible for accessing the randomization system in order to determine patient allocation.

Intervention

Transition program (intervention group)

This study implements a multicomponent intervention, adapted from a brief transition program developed by Hilderson.[23] Adaptation of the program followed the intervention mapping process.[24] Intervention mapping is a thorough method used to build comprehensive programs for health promotion and consists of 6 consecutive steps: 1) assessment of the needs of the target population; 2) defining program objectives by specifying performance and change objectives; 3) determining theory-based intervention methods and practical strategies; 4) designing and producing the intervention program; 5) adoption and implementation of the intervention; and 6) developing and executing a plan for evaluation.[24] Applying the intervention mapping method in this study ensures that the intervention is well-grounded and

For peer review only - http://bmjopen.bmj.com/site/about/guidelines.xhtml

includes the necessary components to target the program objectives. A detailed description of each of the steps will be given in a related article.

The transition program includes 8 key components: a) a transition coordinator (TC); b) a written person-centered transition plan; c) provision of information and education about the condition, treatment and health behaviors; d) availability by telephone and email; e) information about and contact with the GUCH clinic (Grown-Up Congenital Heart Disease); f) guidance of parents; g) meeting with peers; and h) the actual transfer to the GUCH clinic.

These components are implemented in five steps: 1) a first patient visit with the TC; 2) second outpatient visit with TC; 3) information day for adolescents and their parents; 4) third outpatient visit with TC; and 5) actual transfer to the GUCH clinic. The TCs are specialized nurses at the outpatient clinic of pediatric cardiology that have received specific training in performing this intervention.

Throughout the intervention the TCs employ a person-centered care (PCC) approach. PCC implies that participants are active agents in their care and decision-making process.[12] Ekman proposes that PCC can be implemented in three stages.[12] First, initiating the partnership by allowing the patients to tell their story (patient narratives). This allows for the patients' views to be at the center of care. Second, developing the partnership through constant communication between the patient and healthcare providers. This is accomplished by sharing experiences and building a common understanding of the care plan. Third, safeguarding the partnership by documenting the decisions made and the preferences of the patients.

The content of the visits is individualized, age and developmentally adapted and it is documented in the individualized transfer plan. The TCs together with the adolescent are in

For peer review only - http://bmjopen.bmj.com/site/about/guidelines.xhtml

charge of determining which topics are important to discuss, in establishing goals related to patient empowerment and in assessing the need for referral to other services.

Usual care (intervention, control and comparison group)

All adolescents who participate in the study receive usual care, irrespective of whether in the intervention, comparison or control group. Usual care includes visits to an outpatient pediatric cardiology clinic where they meet a nurse and a pediatric cardiologist. Disease complexity determines the frequency of the visits. None of the participating centers have a TC as a part of usual care and there is no documentation of transition plans. It is common practice in Sweden that patients are transferred to adult care around the age of 18 years.

Since usual care can vary across clinics and over time, an assessment of transition-related practices will be done in all the centers at the beginning of the intervention and at the end of the study. This assessment will be comprised of three elements: 1) a structured survey called CHAT (Congenital Heart Adolescent and Transition) (on file); 2) interviews with healthcare professionals who are involved in the care for adolescents with CHD in the participating centers; and 3) observations of the routine during the clinic visits.

Blinding

Due to the nature of the intervention and the study design, it is not possible to blind the participants, TCs or data collection officers (DCOs). However, the TCs and DCOs in charge of carrying out the intervention and data collection are not involved in the preparation of the intervention design or in the statistical analyses.

For peer review only - http://bmjopen.bmj.com/site/about/guidelines.xhtml

To avoid cross contamination between the comparison and the intervention group within the RCT centers, TCs are not involved in the delivery of care of the comparison group and other healthcare professionals in the participating centers will not be informed of the intervention components. DCOs in the control group do not participate in discussions of the intervention.

Intervention fidelity

To assure that the transition program is implemented according to the developed intervention, the TCs are selected based on their experience of working with children and adolescents with CHD. In preparation for this study, they received a specifically designed 4-days training program, addressing adolescents' health and development; communicating with and interviewing young persons; patient education; person-centered care; and the detection of and screening for risk behaviors. Training is given by experts in the aforementioned fields and features different pedagogical approaches, e.g. group discussions, case assessments, and role play. In addition, the TCs were asked to read two textbooks on adolescent health and person-centered care.

Standard Operating Procedures (SOPs) for each group of the study were developed in order to ensure consistency throughout the study. These SOPs included: ethical approval; study preparation; study administration; participants recruitment and survey completion; intervention characteristics; usual care; data storage, entry and security; study progress; and publication policy.

Intervention fidelity is monitored as part of the process evaluation. The Medical Research Council (MRC) guidance on process evaluation of complex interventions is applied,[25] and both quantitative and qualitative methods will be used. In the quantitative

For peer review only - http://bmjopen.bmj.com/site/about/guidelines.xhtml

assessment we investigate the acceptability, adherence/fidelity and attrition, as well as potential safety issues. These data are collected using three assessment forms (enrolment/follow-up form, adverse events report and intervention implementation form) filled out by the TCs during the intervention period. The qualitative assessment will obtain information about the experiences of and implementation by the TCs and will provide insights into the mechanisms of impact based on the experiences of patients and their parents. The process evaluation will be undertaken as a separate study conducted in parallel with this effectiveness study.

Follow-up period and data collection

During the 2-year follow-up period, outcome measurements are assessed on three different occasions: T0, baseline when the participants are 16 years old; T1, midterm when the participants are 17 years; and T2, final data collection when the participants have been transferred to adult care and are approximately 18.5-19 years old (See Figure 1).

Participants from the intervention group will be mailed the set of questionnaires one month before the outpatient clinic visit. If the questionnaires have not been received by the time of the visit, they will have the opportunity to complete them while waiting for their appointment. Patients from the control and comparison groups may not have a scheduled outpatient visit. Therefore, data collection in these groups is completely undertaken by post when the participants are 16 (T0), 17 (T1) and 18.5 years old (T2).

To minimize non-response in the comparison and control groups a modified Dillman procedure will be used.[26] Two weeks after the documents are sent, non-responders will receive a first personalized reminder in the form of a text message. If after five weeks the questionnaires have not been received a reminder letter and a new set of questionnaires is

For peer review only - http://bmjopen.bmj.com/site/about/guidelines.xhtml

sent. After seven weeks, persistent non-responders are contacted by telephone and asked whether they received the questionnaires, if the mailing address is correct, if they want a new set of questionnaires and if they want to continue participating in the study. After 9 weeks a last reminder is sent for those who agree to participate in the study during the telephone call.

Outcome measurements

A background information questionnaire is included, comprising sociodemographic variables such as sex, age, educational level, size of community, place of birth, number of siblings, birth order, living situation, other health conditions and medication (obtained from self-report and medical file).

Standardized questionnaires are used to assess primary and secondary outcomes. Table 1 presents an overview of the characteristics and psychometric properties of the questionnaires used.

For peer review only

For peer review only - http://bmjopen.bmj.com/site/about/guidelines.xhtml

Table 1: Psychometric properties of the questionnaires used in STEPSTONES-ConHD

Variable	Subject	Measurements	Items	Validity	Reliability	Responsiveness	Interpretation
Primary Outcome							
Patient empowerment	AD	Gothenburg Young Persons Empowerment Scale (GYPES)	15	Construct validity supported an adequate model fit of a five factor solution (*df*: 80; $\chi2$: 154-948, p<0.0001; CFI: 0.916; RMSEA: 0.068; SRMR: 0.069) (data on file)<br>Face validity confirmed in young persons with chronic conditions (data on file)	Internal consistency confirmed in young persons with CHD, α=0.819 (data on file)	Floor scores: 0%; Ceiling scores: 1.5% (data on file)	Score from 15-75. Higher score reflecting a higher level of patient empowerment
Secondary Outcomes							
Transition readiness	AD	Readiness for Transition Questionnaire (RTQ) adolescent version	26	Validity based on relationships with other variables confirmed in young persons with kidney transplant, [27] p.91-92	Internal consistency confirmed in young persons with kidney transplant; α values over 0.70,[27]	NR	Scores from 10-40. Higher scores denote increased adolescent or caregiver responsibility
	PA	Readiness for Transition Questionnaire (RTQ) parent version		Construct validity reported for parents of young persons with kidney transplant, [27] p.91-92	Reliability reported for parents of young persons with kidney transplant; α values over 0.70, [27] p.90	NR	

For peer review only - http://bmjopen.bmj.com/site/about/guidelines.xhtml

Knowledge on ConHD	AD	Knowledge Scale for Adults with Congenitally Malformed Hearts (KnoCoMH)	19	Validity was confirmed in adults with CHD in relation to discrimination ability, the relationship of the items and the construct of interest, [28] p.232-234<br><br>KnoCoMH is based on the Leuven Knowledge Scale which has been validated in young persons with CHD, [29] p.79-80	Internal consistency and test-retest reliability confirmed,[28] p.232-234	NR	Scores are calculated by dichotomizing the answers (correct/incorrect) for each domain
Health behaviors	AD	Health Behavior Scale-Congenital Heart Disease (HBS-CHD)	15*	Item content validity, scale content validity index and validity based on relationships to other variables confirmed in adolescents with CHD, [30] p.548	Stability not confirmed.[29]	Confirmed in adolescents with CHD by Guyatt's Responsiveness Index, [30] p.551	Substance use, dental hygiene and total health risk score from 0-100. Physical exercise score from 0-∞
Patient-reported health		Pediatric Quality of Life Inventory 4.0 (PedsQL) generic module	23	Convergent validity, measurement invariance and factor structure confirmed in pediatric populations,<br><br>[31] p.336-337<br><br>[32] p.1507-1510	Test-retest reliability, and internal consistency show α values over 0.70 in pediatric populations, [32] p.1507<br><br>[31] p.333-335	Minimal clinically important differences reported for young people with CHD was 6.0 for the total score,[33]	Scores from 0 to 100. Higher scores indicate a better perceived health status
	AD	Pediatric Quality of Life Inventory 4.0 (PedsQL) cardiac module	27	Convergent validity was confirmed, [34] p.215	Internal consistency for majority of scales exceeded α values of 0.70, [34] p.214	Minimal clinically important differences reported for young people with CHD ranged from 7.6	Scores from 0 to 100. Higher scores indicate lower problems

For peer review only - http://bmjopen.bmj.com/site/about/guidelines.xhtml

						for symptoms to 12.6 for communication, [33]	
		EQ-5D-3L	5	Convergent and discriminative validity confirmed in cardiovascular patients, [35] p.6	Test-retest reliability confirmed in cardiovascular patients, [35] p.6	Confirmed in cardiovascular patients, [35] p.6	Scores from 1 (no problems) to 3 (extreme problems) on five dimensions.<br><br>Scores from 0 (worst imaginable health state) to 100 (best imaginable health state)
Quality of life	AD	Linear analog scale (LAS)	1	Validity based on relationship with Satisfaction with Life Scale found to be highly correlated (ρ=0.52). Test content confirmed in adults with CHD (100% of patients understood the wording and format), [36]<br><br>Validity has been evaluated in adolescents with CHD [37, 38]	Test-retest reliability confirmed in adults with CHD (ICC=0.65), [36]	Confirmed in adults with CHD (Floor scores= 0%, ceiling scores=2.7%), [36]	Score is from 100 (best imaginable health status) to 0 (worst imaginable health status)
Healthcare utilization	AD	Healthcare utilization questionnaire	11	NA	NA	NA	NA
Parental uncertainty towards transfer to adult care	PA	Linear analog scale (LAS)	1	Face validity confirmed in parents of young persons with chronic conditions (data on file).<br><br>Test content confirmed in	NR	Floor scores: 7.8%; Ceiling scores: 4.8% (data on file)	Score is from 0 (very uncertain about the transfer to adult care) to 100 (not uncertain

For peer review only - http://bmjopen.bmj.com/site/about/guidelines.xhtml

				parents of young persons with CHD (100% of patients understood the wording and format).			about the transfer of care)

AD: adolescents. PA: parents. NA: not applicable. NR: not reported. CFI: comparative fit index. RMSEA: root mean square error of approximation. SRMR: standardized root mean square residual. $\chi2$: chi-square test of model fit.

For peer review only

Primary outcome measures

The primary outcome is patient empowerment, defined as "*an enabling process or outcome arising from communication with the healthcare professional and a mutual sharing of resources over information relating to illness, which enhances the patient's feelings of control, self-efficacy, coping abilities and ability to achieve change over their condition.*[9] Empowerment includes five dimensions: 1) identity; 2) knowledge and understanding; 3) personal control; 4) decision making; and 5) enabling others.[9]

Since no questionnaire to measure the level of empowerment in young persons with chronic conditions exists, we developed the Gothenburg Young Persons Empowerment Scale-Congenital Heart Disease module (GYPES-CHD). The scale has 15 items, 3 items for each of the aforementioned dimensions. Each item is answered on a five-point Likert scale: strongly disagree; disagree; neither agree or disagree; agree; and strongly agree. A total score is calculated by summing the item scores. The total score ranges from 15-75 points, with a higher score reflecting a higher level of patient empowerment.

Face validity was confirmed after cognitive interviews with 6 young persons with CHD. The psychometric properties of this scale were examined in a cross-sectional study in 205 young persons with CHD. Internal consistency of the items was confirmed with a Cronbach's alpha of 0.821. No floor or ceiling effect in scoring was observed. A complete description of the psychometric evaluation of GYPES will be provided in another article.

Secondary outcomes measures

We will measure six secondary outcomes in the adolescents: transition readiness, health behaviors, knowledge about CHD, healthcare utilization, quality of life and patient-reported health. In the parents, we will measure transition readiness (proxy) and parental uncertainty toward transition.

For peer review only - http://bmjopen.bmj.com/site/about/guidelines.xhtml

Transition readiness

Transition readiness is defined as the "*adolescent's readiness to assume complete responsibility for their healthcare and their readiness to transfer to adult medical care*".[27] Transition readiness is evaluated using the Readiness for Transition Questionnaire (RTQ), adolescent and parent versions. These questionnaires examine two aspects. First the overall transition readiness is assessed, using two items ranging from 1-4. The sum of these two items results in a score ranging from 2-8. Second, the frequency of adolescent responsibility and parental involvement are reported in ten different health behaviors, on a five-point Likert. Each variable results in a total score ranging from 10 to 40, higher scores indicate higher adolescent responsibility or parental involvement, respectively.

Knowledge about CHD

Adolescents' knowledge about their CHD is assessed with the Knowledge Scale for adults with Congenital Malformed Hearts (KnoCoMH). [28] This instrument evaluates the level of knowledge within four domains: disease and its treatment; complications prevention; physical activity; and reproductive issues.[28] The questionnaire includes 19 items and scores are calculated by dichotomizing answers as correct or incorrect. Access to the medical files is needed in order to confirm the answers in some of the items. For participants where medical treatment or endocarditis prophylaxis is not recommended, the domains are excluded.

Health behaviors

Health behaviors are measured with the Health Behavior Scale-CHD. Health behaviors are *"activities a person undertakes to maintain or improve health and prevent diseases"*.[30] This scale assesses alcohol consumption, tobacco use, dental care and physical activity. The scale has 15 items that help to calculate three summary risk scores: substance

For peer review only - http://bmjopen.bmj.com/site/about/guidelines.xhtml

use (score 0-100), dental hygiene (score 0-100) and total health risk score (score 0-100). Higher risk scores represent unhealthier behaviors.

Patient-reported health

Patient-reported health is assessed by the generic and cardiac modules of the Pediatric Quality of Life inventory 4.0 (PedsQL 4.0), as well as the EuroQoL 5 dimensions 3 levels (EQ-5D-3L). The PedsQL 4.0 provides information on health status in relation to psychosocial and physical health.[32] The generic module consists of 23 items that cover four dimensions: physical; emotional; social functioning; and school functioning. Items are answered by using a five-point Likert scale: never, almost never, sometimes, often, and always. For each dimension, a scale score can be calculated, which ranges from 0 to 100. Further, a physical health summary score, a psychosocial health summary score, and a total score can be calculated.

The cardiac module contains 27 items that cover six dimensions: symptoms; treatment; perceived physical appearance; treatment anxiety; cognitive problems; and communication. Each item is answered using a 5-point Likert scale (never, almost never, sometimes, often and always). As in the generic version, scores are transformed on a scale from 0 to 100, and then a mean subscale core is calculated.[34]

The EQ-5D-3L comprises a descriptive system and a visual analog scale (VAS). The descriptive system has 5 dimensions (mobility, self-care, usual activities, pain/discomfort and anxiety/depression) with 3 response categories per dimension.[39] The participants are asked to indicate which statement is most suitable in relation to their health status. The VAS measures the participants' self-rated health on a scale with the endpoints "best imaginable health possible" (=100) and "worst imaginable health state (=0)". The descriptive system allows for the determination of a 5 digit health state that combines the level of perceived problems in

For peer review only - http://bmjopen.bmj.com/site/about/guidelines.xhtml

each dimension (level 1: indicating no problem, level 2: indicating some problems and level 3: indicating extreme problems). Information obtained from the descriptive system can be converted to a single summary index by assigning values to each level of every dimension.

Quality of life

Quality of life is defined as "*the overall degree of life satisfaction that is positively or negatively influenced by individuals' perception of certain aspects of life important to them, including matters both related and unrelated to health"*.[40] This variable is measured using a 10 cm linear analog scale (LAS), [36] where the participants indicate in the scale how they judge their current quality of life. The scale looks similar to the VAS of the EQ-5D, but has the endpoints "best imaginable quality of life" (=100) and "worst imaginable quality of life" (=0).

Healthcare utilization

Healthcare utilization within the preceding six months is measured in relation to their ConHD and other possible conditions. The measurement consists of six dimensions: hospital stay; healthcare contact; specialized care; emergency care; school absence; and follow-up unit after transfer to adult care. This assessment was developed to be used in the APPROACH-IS project.[41] After the transfer to adult care, we will assess the proportion of patients that are in follow-up in a GUCH unit, both in the intervention as control arms.

Parental uncertainty

Parental uncertainty is understood as the degree of certainty/uncertainty the parents have in relation to their children being transferred to adult care. This outcome is measured through LAS with the endpoints "extremely uncertain" (=100) and "not uncertain at all" (=0). This LAS has been developed for the purpose of the present study.

For peer review only - http://bmjopen.bmj.com/site/about/guidelines.xhtml

In addition to the primary and secondary endpoints, we will calculate a composite endpoint. We will do so because the transition program is a complex intervention that may have varying effects on different outcomes. Prior studies on the impact of transition programs showed that the intervention has impacted on transition readiness,[16] patient knowledge,[16] quality of life,[42] and psychosocial health.[42] Therefore, each patient will be categorized as improved or not. Patients will be classified as improved if they have an improvement in empowerment, transition readiness, knowledge, quality of life, or psychosocial health of 0.5 standard deviations compared to their baseline measurement.

Data management

Data are entered into a web-based system by the TC and the DCO. Those with access to the system are the TC, DCO, project coordinator and principal investigator, and each have a personal login. All data are checked for completeness and the quality of the data is monitored at least twice a year by the members of the research group. This process is done by verifying records with the raw data and checking frequency distributions to identify responses outside of the given possibilities.

Statistical analyses plan

The primary analyses will be done following the intention to treat principle which helps to avoid biases in the comparisons between groups. The primary endpoint (level of empowerment) will be analyzed using Fishers non-parametric permutation test unadjusted between the two groups. Complementary between-group analyses will be made using

For peer review only - http://bmjopen.bmj.com/site/about/guidelines.xhtml

ANCOVA adjusted for baseline variables that differ significantly between groups and for each center.

All analyses will be predefined in a comprehensive statistical analysis plan before data lock. The level of significance will be at *p value* equal to or less than 0.05. Two-sided tests will be used.

Trial duration

Patient recruitment began July 2016 and is planned to finish in December 2017. Once recruited, patients are followed-up until they are transferred to GUCH. Data collection is expected to be finished by December 2019. After the data set is locked, analysis and dissemination of results will begin.

Ethical considerations

The study was approved by the Regional Ethical Board of Gothenburg, Sweden (No.931-15). Before inclusion in the study, the TCs and DCOs will seek written informed consent from the adolescents and their parents after they have received written and oral information about the study. Participants have the right to withdraw at any point throughout the study. The principles established in the Declaration of Helsinki will be followed during the entire duration of the study.[43]

To ensure confidentiality personal data is coded. Participant and study-related information is stored in locked cabinets and digital documents are password-protected.

For peer review only - http://bmjopen.bmj.com/site/about/guidelines.xhtml

Follow-up on adverse events

All adverse events (AEs) must be reported to the principal investigator and project coordinator within a week. In case of a serious AE, the report must be made immediately. Appropriate measures will be undertaken in every AE in order to ensure patient safety.

Dissemination

The results of this study will be submitted to peer reviewed journals for publication following the CONSORT statement.[44] Abstracts for poster or oral presentations will be submitted to international conferences. Authorship is granted to a collaborator from every participating center and members of the research group fulfilling the criteria of authorship [45, 46]. If possible, we will include "on behalf of STEPSTONES-CHD" in the list of authors. After completion of the study, the data set will be available upon formal request.

DISCUSSION

Previous studies have tried to determine the effectiveness of transition programs. However, due to several methodological limitations, the true effect of these interventions is still uncertain.[7] Therefore, we will conduct a study that assesses the effectiveness of a structured, person-centered transition program to empower adolescents with CHD in the transition to adulthood. Through the implementation of the proposed transition program, we expect an increase in the level of empowerment. A high level of empowerment can help the adolescent navigate through the adult care system, participate in care planning and increase self-management and decision-making skills.[8]

For peer review only - http://bmjopen.bmj.com/site/about/guidelines.xhtml

The intervention in this trial was designed and initially evaluated by Hilderson,[23, 42] in Belgium. Therefore, it was important to determine if the intervention components were suitable in the Swedish setting. To accomplish this, five preparatory studies were done (four qualitative, one quantitative study). The qualitative studies included individual and focus group interviews with young persons with CHD and their parents.[47, 48] These studies provided an overview of the current transition practices in Sweden and the needs of this population before the transfer to adult care. The quantitative study was a cross-sectional study including 205 young persons with CHD, and served as a pilot-test for our primary outcome measurement. Furthermore, the intervention was discussed with a panel of international experts with experience in transition research in adolescents with chronic conditions and patient representatives.

The preparatory studies and the discussion with the panel of international experts improved the quality, feasibility and acceptability of the intervention. This interdisciplinary and comprehensive approach increases the chance of producing a high-quality intervention with positive results.[49]

The present study has several strengths, because we explicitly take limitations of prior studies into account. First, we employ a RCT which is the gold standard for the assessment of the effectiveness of interventions and provides the highest level of evidence. The stratified block randomization that we undertake ensures that the intervention and comparison groups are balanced and that potential confounding factors, such as complexity of the disease or center characteristics, are equally distributed and therefore minimize the risk of biasing the results.

Second, the proposed hybrid experimental design allows us to account for potential contamination of the control group. Indeed, the 3 arms allow performance of direct

For peer review only - http://bmjopen.bmj.com/site/about/guidelines.xhtml

comparison between the intervention and comparison group while enabling a comparison of findings with those obtained in intervention-naive centers.

Third, patient empowerment is used as the primary outcome. Empowerment is argued to be important for the development and application of skills to promote behavioral changes in the adolescents.[10] Admittedly, empowerment can be seen both as a process and an outcome.[10] However, being an intermediate outcome, empowerment can be argued to be proximal to the intervention whereas continuing cardiac care, reduced morbidity or mortality are probably the ultimate, but more distal outcomes.

Fourth, the PCC approach that is used throughout the transition program is an important asset in the process of empowering patients.[11] Providing transitional care from a person-centered perspective is novel and has not been evaluated before in this age group.

Fifth, we follow-up the patients for more than 2 years. Doing so, we substantially expand the follow-up period of 12 months as used in prior studies.[7]

Sixth, since usual care may vary across centers and over time, we carry out a formal usual care assessment. Such a usual care assessment is important to draw firm conclusions and yet this is seldom performed in clinical trials.[50]

Irrespective of these strengths, we anticipate some limitations as well. First, we are not able to blind the participants or the healthcare team that will perform the intervention. To minimize a potential impact, neither the therapeutic team nor the TCs participate in the data analysis. Second, there is a possibility of a relatively long period of recruitment for participants which can require extending the trial period and delaying data analysis. Third, transition interventions have been advised to start early on, e.g. between 10-12 years of age, and have a long follow-up.[51] Furthermore, a long follow-up allows measuring continuity of care in this population and provide insight on the effects of the transition program over this

For peer review only - http://bmjopen.bmj.com/site/about/guidelines.xhtml

parameter. Although we recognize the importance of starting early, it would have jeopardized the feasibility of the study, because it would have taken almost a decade to finish recruitment and follow-up until the age of 18 years. Nonetheless, it could be possible to later on, assess loss of follow-up in a separate study

Conclusion

This article described the study protocol for a study to assess the effectiveness of a structured, person-centered transition program for young persons with CHD. This study accounts for limitations of previous research and addresses an existing gap in the evidence of transitional care. It is hoped the findings will provide strong evidence of the effectiveness of this intervention in young people with CHD. If successful, this transition program could potentially be implemented as part of routine care provided to young persons with CHD, and transfer to other chronic conditions can be explored.

Trial status

Status of the trial at the time of submission: patient recruitment

List of abbreviations

CHD: congenital heart disease

DCO: data collection officer

GYPES: Gothenburg Young Persons Empowerment Scale

For peer review only - http://bmjopen.bmj.com/site/about/guidelines.xhtml

GYPES-CHD: Gothenburg Young Persons Empowerment Scale-Congenital Heart Disease module

GUCH: grown-up congenital heart disease unit

MRC: Medical Research Council

RCT: randomized controlled trial

STEPTONES: Swedish Transition Effects Project Supporting Teenagers with chrONic mEdical conditionS

TC: transition coordinator

Competing interests

There are no conflicts of interests.

Funding

This study is supported by research grants from the Swedish Heart-Lung Foundation; Swedish Research Council for Health, Working Life and Welfare; Swedish Children Heart Association; Swedish Research Council; and the Institute of Health and Care Sciences of the University of Gothenburg.

Author's contributions

For peer review only

MAM, CSL, ELB and PM conceived the study, were responsible for the design of the study protocol and drafted the manuscript. All authors critically revised and approved the manuscript.

Author's information

MAM is a PhD student at the Institute of Health and Care Sciences, University of Gothenburg, Sweden and at the Academic Center for Nursing and Midwifery, Department of Public Health and Primary Care, KU Leuven, Belgium. CSL is senior lecturer at the Institute of Health and Care Sciences, University of Gothenburg, Sweden. ELB is senior lecturer at the Institute of Health and Care Sciences, University of Gothenburg, Sweden and a pediatric nurse in Queen Silvia Children's Hospital. PM is Professor of Healthcare and Nursing Science and Program Director at the KU Leuven Department of Public Health and Primary Care, Belgium and Visiting Professor at the Institute of Health and Care Sciences, University of Gothenburg, Sweden.

Acknowledgements

We would like to acknowledge the collaboration from the members of the panel of international experts and of the Advisory Board.

References

1. Kennedy A, Sloman F, Douglass JA, et al. Young people with chronic illness: the approach to transition. Intern Med J 2007;**37**(8):555-60.

For peer review only - http://bmjopen.bmj.com/site/about/guidelines.xhtml

2. Moons P, Bovijn L, Budts W, et al. Temporal trends in survival to adulthood among patients born with congenital heart disease from 1970 to 1992 in Belgium. Circulation 2010;**122**(22):2264-72.

3. Meadows AK, Bosco V, Tong E, et al. Transition and transfer from pediatric to adult care of young adults with complex congenital heart disease. Curr Cardiol Rep 2009;**11**(4):291-97.

4. American Academy of Pediatrics. A consensus statement on health care transitions for young adults with special health care needs. Pediatrics 2002;**110**(6 Pt 2):1304-6.

5. Warnes CA, Williams RG, Bashore TM, et al. ACC/AHA 2008 Guidelines for the Management of Adults With Congenital Heart Disease: A Report of the American College of Cardiology/American Heart Association Task Force on Practice Guidelines (Writing Committee to Develop Guidelines on the Management of Adults With Congenital Heart Disease): Developed in Collaboration With the American Society of Echocardiography, Heart Rhythm Society, International Society for Adult Congenital Heart Disease, Society for Cardiovascular Angiography and Interventions, and Society of Thoracic Surgeons. Circulation 2008;**118**(23):E714-E833.

6. Crowley R, Wolfe I, Lock K, et al. Improving the transition between paediatric and adult healthcare: a systematic review. Arch Dis Child 2011;**96**(6):548-53.

7. Campbell F, Biggs K, Aldiss SK, et al. Transition of care for adolescents from paediatric services to adult health services. Cochrane Database Syst Rev 2016;**4**:CD009794.

8. Bravo P, Edwards A, Barr PJ, et al. Conceptualising patient empowerment: a mixed methods study. BMC Health Serv Res 2015;**15**:252.

9. Small N, Bower P, Chew-Graham CA, et al. Patient empowerment in long-term conditions: development and preliminary testing of a new measure. BMC Health Serv Res 2013;**13**:263.

10. Anderson RM, Funnell MM. Patient empowerment: Myths and misconceptions. Patient Educ Couns 2010;**79**(3):277-82.

11. Holmstrom I, Roing M. The relation between patient-centeredness and patient empowerment: a discussion on concepts. Patient Educ Couns 2010;**79**(2):167-72.

12. Ekman I, Swedberg K, Taft C, et al. Person-centered care--ready for prime time. Eur J Cardiovasc Nurs 2011;**10**(4):248-51.

For peer review only

13. Dolk H, Loane M, Garne E, et al. Congenital heart defects in Europe: prevalence and perinatal mortality, 2000 to 2005. Circulation 2011;**123**(8):841-9.

14. Marelli AJ, Ionescu-Ittu R, Mackie AS, et al. Lifetime prevalence of congenital heart disease in the general population from 2000 to 2010. Circulation 2014;**130**(9):749-56.

15. Hilderson D, Saidi AS, Van Deyk K, et al. Attitude toward and current practice of transfer and transition of adolescents with congenital heart disease in the United States of America and Europe. Pediatr Cardiol 2009;**30**(6):786-93.

16. Mackie AS, Islam S, Magill-Evans J, et al. Healthcare transition for youth with heart disease: a clinical trial. Heart 2014;**100**(14):1113-8.

17. Goossens E, Fieuws S, Van Deyk K, et al. Effectiveness of structured education on knowledge and health behaviors in patients with congenital heart disease. J Pediatr 2015;**166**(6):1370-6 e1.

18. Goossens E, Van Deyk K, Zupancic N, et al. Effectiveness of structured patient education on the knowledge level of adolescents and adults with congenital heart disease. Eur J Cardiovasc Nurs 2014;**13**(1):63-70.

19. Chan AW, Tetzlaff JM, Altman DG, et al. SPIRIT 2013 statement: defining standard protocol items for clinical trials. Ann Intern Med 2013;**158**(3):200-7.

20. Mitchell SC, Korones SB, Berendes HW. Congenital heart disease in 56,109 births. Incidence and natural history. Circulation 1971;**43**(3):323-32.

21. Efird J. Blocked randomization with randomly selected block sizes. Int J Environ Res Public Health 2011;**8**(1):15-20.

22. Warnes CA, Liberthson R, Danielson GK, et al. Task force 1: the changing profile of congenital heart disease in adult life. J Am Coll Cardiol 2001;**37**(5):1170-5.

23. Hilderson D, Westhovens R, Wouters C, et al. Rationale, design and baseline data of a mixed methods study examining the clinical impact of a brief transition programme for young people with juvenile idiopathic arthritis: the DON'T RETARD project. BMJ Open 2013;**3**(12):e003591.

24. Bartholomew LK, Markham CM, Ruiter R, et al. *Planning health promotion programs : an intervention mapping approach*. 4th ed. San Francisco: Jossey-Bass, 2016.

For peer review only - http://bmjopen.bmj.com/site/about/guidelines.xhtml

25. Moore GF, Audrey S, Barker M, et al. Process evaluation of complex interventions: Medical Research Council guidance. BMJ 2015;**350**:h1258.

26. Dillman D. Mail and other self-administered questionnaires. In: Rossi PH, Wright JD, Anderson AB, eds. Handbook of survey research. New York: Academic Press, 1983:pp. 359–77.

27. Gilleland J, Amaral S, Mee L, et al. Getting ready to leave: transition readiness in adolescent kidney transplant recipients. J Pediatr Psychol 2012;**37**(1):85-96.

28. Ronning H, Arestedt K, Erik Nielsen N, et al. Development and psychometric evaluation of the knowledge scale for adults with congenitally malformed hearts. J Cardiovasc Nurs 2013;**28**(3):228-37.

29. Yang HL, Chen YC, Wang JK, et al. Measuring knowledge of patients with congenital heart disease and their parents: validity of the 'Leuven Knowledge Questionnaire for Congenital Heart Disease'. *Eur J Cardiovasc Nurs* 2012;**11**(1):77-84. doi: 10.1177/1474515111429662

30. Goossens E, Luyckx K, Mommen N, et al. Health risk behaviors in adolescents and emerging adults with congenital heart disease: psychometric properties of the Health Behavior Scale-Congenital Heart Disease. Eur J Cardiovasc Nurs 2013;**12**(6):544-57.

31. Varni JW, Burwinkle TM, Seid M, et al. The PedsQL™* 4.0 as a Pediatric Population Health Measure: Feasibility, Reliability, and Validity. Ambul Pediatr 2003;**3**(6):329-41.

32. Petersen S, Hagglof B, Stenlund H, et al. Psychometric properties of the Swedish PedsQL, Pediatric Quality of Life Inventory 4.0 generic core scales. Acta Paediatr 2009;**98**(9):1504-12.

33. Bratt E-L, Luyckx K, Goossens E, et al. Patient-Reported Health in Young People With Congenital Heart Disease Transitioning to Adulthood. J Adolesc Health 2015;**57**(6):658-65.

34. Sand P, Kljajić M, Sunnegårdh J. The reliability of the Pediatric Quality of Life Inventory 3.0 Cardiac Module™ for Swedish children with congenital heart defects. Nordic Psychology 2013;**65**(3):210-23.

35. Dyer MT, Goldsmith KA, Sharples LS, et al. A review of health utilities using the EQ-5D in studies of cardiovascular disease. Health and Quality of Life Outcomes 2010;**8**(1):1-12.

36. Moons P, Van Deyk K, De Bleser L, et al. Quality of life and health status in adults with congenital heart disease: a direct comparison with healthy counterparts. Eur J Cardiovasc Prev Rehabil 2006;**13**(3):407-13.

For peer review only - http://bmjopen.bmj.com/site/about/guidelines.xhtml

37. Luyckx K, Missotten L, Goossens E, et al. Individual and contextual determinants of quality of life in adolescents with congenital heart disease. *J Adolesc Health* 2012;**51**(2):122-8. doi: 10.1016/j.jadohealth.2011.11.007

38. Apers S, Moons P, Goossens E, et al. Sense of coherence and perceived physical health explain the better quality of life in adolescents with congenital heart disease. *Eur J Cardiovasc Nurs* 2013;**12**(5):475-83. doi: 10.1177/1474515113477955

39. EuroQoL Group. EuroQol--a new facility for the measurement of health-related quality of life. Health Policy 1990;**16**(3):199-208.

40. Moons P, Marquet K, Budts W, et al. Validity, reliability and responsiveness of the "Schedule for the Evaluation of Individual Quality of Life – Direct Weighting" (SEIQoL-DW) in congenital heart disease. Health and Quality of Life Outcomes 2004;**2**(1):1-8.

41. Apers S, Kovacs AH, Luyckx K, et al. Assessment of Patterns of Patient-Reported Outcomes in Adults with Congenital Heart disease - International Study (APPROACH-IS): rationale, design, and methods. Int J Cardiol 2015;**179**:334-42.

42. Hilderson D, Moons P, Van der Elst K, et al. The clinical impact of a brief transition programme for young people with juvenile idiopathic arthritis: results of the DON'T RETARD project. Rheumatology (Oxford) 2016;**55**(1):133-42.

43. World Medical Association. World medical association declaration of helsinki: Ethical principles for medical research involving human subjects. JAMA 2013;**310**(20):2191-94.

44. Moher D, Hopewell S, Schulz KF, et al. CONSORT 2010 explanation and elaboration: updated guidelines for reporting parallel group randomised trials. BMJ 2010;**340**:c869.

45. Bourbonniere MC, Russell DJ, Goldsmith CH. Authorship issues: one research center's experience with developing author guidelines. Am J Occup Ther 2006;**60**(1):111-7.

46. Whellan DJ, Ellis SJ, Kraus WE, et al. Method for establishing authorship in a multicenter clinical trial. Ann Intern Med 2009;**151**(6):414-20.

47. Asp A, Bratt EL, Bramhagen AC. Transfer to Adult Care--Experiences of Young Adults with Congenital Heart Disease. J Pediatr Nurs 2015;**30**(5):e3-10.

For peer review only

For peer review only - http://bmjopen.bmj.com/site/about/guidelines.xhtml

48. Burstrom A, Ojmyr-Joelsson M, Bratt EL, et al. Adolescents With Congenital Heart Disease and Their Parents: Needs Before Transfer to Adult Care. J Cardiovasc Nurs 2015.

49. Craig P, Dieppe P, Macintyre S, et al. Developing and evaluating complex interventions: the new Medical Research Council guidance. BMJ : British Medical Journal 2008;**337**:a1655.

50. Erlen JA, Tamres LK, Reynolds N, et al. Assessing usual care in clinical trials. West J Nurs Res 2015;**37**(3):288-98.

51. Sable C, Foster E, Uzark K, et al. Best practices in managing transition to adulthood for adolescents with congenital heart disease: the transition process and medical and psychosocial issues: a scientific statement from the American Heart Association. Circulation 2011;**123**(13):1454-85

For peer review only

For peer review only - http://bmjopen.bmj.com/site/about/guidelines.xhtml

Figure legends

Figure 1: Overview of the study design and implementation steps

For peer review only

For peer review only - http://bmjopen.bmj.com/site/about/guidelines.xhtml

Figure 1: Overview of study design and implementation steps

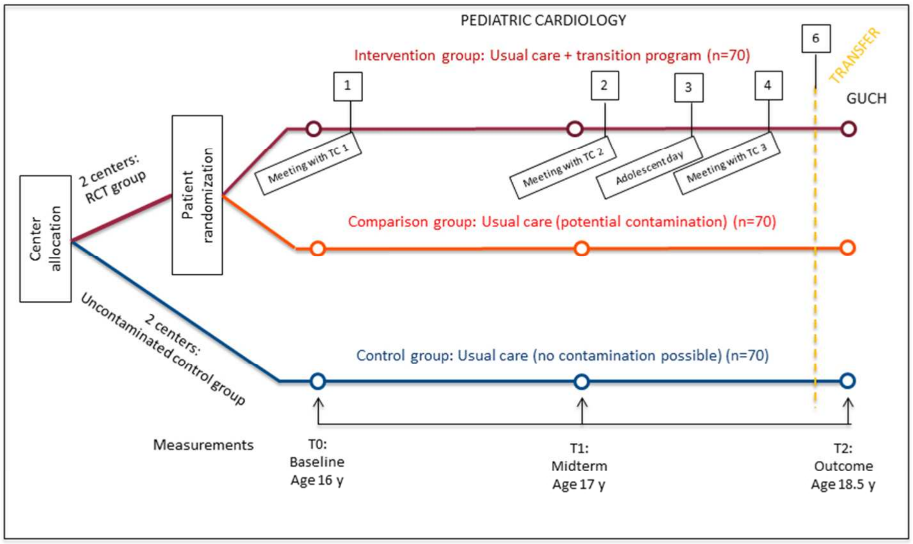

RCT: randomized controlled trial; TC: transition coordinator; GUCH: grown-up congenital heart disease clinic

81x60mm (300 x 300 DPI)

SPIRIT

STANDARD PROTOCOL ITEMS: RECOMMENDATIONS FOR INTERVENTIONAL TRIALS

SPIRIT 2013 Checklist: Recommended items to address in a clinical trial protocol and related documents*

Section/item	Item No	Description	Addressed on page number
**Administrative information**			
Title	1	Descriptive title identifying the study design, population, interventions, and, if applicable, trial acronym	1
Trial registration	2a	Trial identifier and registry name. If not yet registered, name of intended registry	3
	2b	All items from the World Health Organization Trial Registration Data Set	NA
Protocol version	3	Date and version identifier	NA
Funding	4	Sources and types of financial, material, and other support	28~~7~~
Roles and responsibilities	5a	Names, affiliations, and roles of protocol contributors	29~~7~~
	5b	Name and contact information for the trial sponsor	NA
	5c	Role of study sponsor and funders, if any, in study design; collection, management, analysis, and interpretation of data; writing of the report; and the decision to submit the report for publication, including whether they will have ultimate authority over any of these activities	NA
	5d	Composition, roles, and responsibilities of the coordinating centre, steering committee, endpoint adjudication committee, data management team, and other individuals or groups overseeing the trial, if applicable (see Item 21a for data monitoring committee)	NA

For peer review only - http://bmjopen.bmj.com/site/about/guidelines.xhtml

For peer review only

**Introduction**			
Background and rationale	6a	Description of research question and justification for undertaking the trial, including summary of relevant studies (published and unpublished) examining benefits and harms for each intervention	4-5
	6b	Explanation for choice of comparators	4-5
Objectives	7	Specific objectives or hypotheses	5-6
Trial design	8	Description of trial design including type of trial (eg, parallel group, crossover, factorial, single group), allocation ratio, and framework (eg, superiority, equivalence, noninferiority, exploratory)	6
**Methods: Participants, interventions, and outcomes**			
Study setting	9	Description of study settings (eg, community clinic, academic hospital) and list of countries where data will be collected. Reference to where list of study sites can be obtained	6
Eligibility criteria	10	Inclusion and exclusion criteria for participants. If applicable, eligibility criteria for study centres and individuals who will perform the interventions (eg, surgeons, psychotherapists)	7
Interventions	11a	Interventions for each group with sufficient detail to allow replication, including how and when they will be administered	8-10
	11b	Criteria for discontinuing or modifying allocated interventions for a given trial participant (eg, drug dose change in response to harms, participant request, or improving/worsening disease)	NA
	11c	Strategies to improve adherence to intervention protocols, and any procedures for monitoring adherence (eg, drug tablet return, laboratory tests)	11
	11d	Relevant concomitant care and interventions that are permitted or prohibited during the trial	9-10
Outcomes	12	Primary, secondary, and other outcomes, including the specific measurement variable (eg, systolic blood pressure), analysis metric (eg, change from baseline, final value, time to event), method of aggregation (eg, median, proportion), and time point for each outcome. Explanation of the clinical relevance of chosen efficacy and harm outcomes is strongly recommended	13~~2~~-2~~0~~2
Participant timeline	13	Time schedule of enrolment, interventions (including any run-ins and washouts), assessments, and visits for participants. A schematic diagram is highly recommended (see Figure)	12, (see Figure 1)

For peer review only - http://bmjopen.bmj.com/site/about/guidelines.xhtml

Sample size	14	Estimated number of participants needed to achieve study objectives and how it was determined, including clinical and statistical assumptions supporting any sample size calculations	6-7
Recruitment	15	Strategies for achieving adequate participant enrolment to reach target sample size	~~NR~~12

Methods: Assignment of interventions (for controlled trials)

Allocation:

Sequence generation	16a	Method of generating the allocation sequence (eg, computer-generated random numbers), and list of any factors for stratification. To reduce predictability of a random sequence, details of any planned restriction (eg, blocking) should be provided in a separate document that is unavailable to those who enrol participants or assign interventions	7
Allocation concealment mechanism	16b	Mechanism of implementing the allocation sequence (eg, central telephone; sequentially numbered, opaque, sealed envelopes), describing any steps to conceal the sequence until interventions are assigned	7
Implementation	16c	Who will generate the allocation sequence, who will enrol participants, and who will assign participants to interventions	~~7~~Randomization section: 7-8
Blinding (masking)	17a	Who will be blinded after assignment to interventions (eg, trial participants, care providers, outcome assessors, data analysts), and how	10
	17b	If blinded, circumstances under which unblinding is permissible, and procedure for revealing a participant's allocated intervention during the trial	NA

Methods: Data collection, management, and analysis

Data collection methods	18a	Plans for assessment and collection of outcome, baseline, and other trial data, including any related processes to promote data quality (eg, duplicate measurements, training of assessors) and a description of study instruments (eg, questionnaires, laboratory tests) along with their reliability and validity, if known. Reference to where data collection forms can be found, if not in the protocol	12, 14-18~~6~~
	18b	Plans to promote participant retention and complete follow-up, including list of any outcome data to be collected for participants who discontinue or deviate from intervention protocols	12

Data management	19	Plans for data entry, coding, security, and storage, including any related processes to promote data quality (eg, double data entry; range checks for data values). Reference to where details of data management procedures can be found, if not in the protocol	22~~1~~
Statistical methods	20a	Statistical methods for analysing primary and secondary outcomes. Reference to where other details of the statistical analysis plan can be found, if not in the protocol	2~~1~~2-23
	20b	Methods for any additional analyses (eg, subgroup and adjusted analyses)	22-23~~1~~
	20c	Definition of analysis population relating to protocol non-adherence (eg, as randomised analysis), and any statistical methods to handle missing data (eg, multiple imputation)	NR
**Methods: Monitoring**			
Data monitoring	21a	Composition of data monitoring committee (DMC); summary of its role and reporting structure; statement of whether it is independent from the sponsor and competing interests; and reference to where further details about its charter can be found, if not in the protocol. Alternatively, an explanation of why a DMC is not needed	NA
	21b	Description of any interim analyses and stopping guidelines, including who will have access to these interim results and make the final decision to terminate the trial	NA
Harms	22	Plans for collecting, assessing, reporting, and managing solicited and spontaneously reported adverse events and other unintended effects of trial interventions or trial conduct	2~~2~~4
Auditing	23	Frequency and procedures for auditing trial conduct, if any, and whether the process will be independent from investigators and the sponsor	NA
**Ethics and dissemination**			
Research ethics approval	24	Plans for seeking research ethics committee/institutional review board (REC/IRB) approval	23~~1-22~~
Protocol amendments	25	Plans for communicating important protocol modifications (eg, changes to eligibility criteria, outcomes, analyses) to relevant parties (eg, investigators, REC/IRBs, trial participants, trial registries, journals, regulators)	NA

For peer review only - http://bmjopen.bmj.com/site/about/guidelines.xhtml

Consent or assent	26a	Who will obtain informed consent or assent from potential trial participants or authorised surrogates, and how (see Item 32)	~~22~~21
	26b	Additional consent provisions for collection and use of participant data and biological specimens in ancillary studies, if applicable	NA
Confidentiality	27	How personal information about potential and enrolled participants will be collected, shared, and maintained in order to protect confidentiality before, during, and after the trial	23~~2~~
Declaration of interests	28	Financial and other competing interests for principal investigators for the overall trial and each study site	28~~6~~
Access to data	29	Statement of who will have access to the final trial dataset, and disclosure of contractual agreements that limit such access for investigators	~~NR~~22
Ancillary and post-trial care	30	Provisions, if any, for ancillary and post-trial care, and for compensation to those who suffer harm from trial participation	NA
Dissemination policy	31a	Plans for investigators and sponsor to communicate trial results to participants, healthcare professionals, the public, and other relevant groups (eg, via publication, reporting in results databases, or other data sharing arrangements), including any publication restrictions	24~~2~~
	31b	Authorship eligibility guidelines and any intended use of professional writers	24~~2~~
	31c	Plans, if any, for granting public access to the full protocol, participant-level dataset, and statistical code	NA
**Appendices**			
Informed consent materials	32	Model consent form and other related documentation given to participants and authorised surrogates	Available upon request
Biological specimens	33	Plans for collection, laboratory evaluation, and storage of biological specimens for genetic or molecular analysis in the current trial and for future use in ancillary studies, if applicable	NA

*It is strongly recommended that this checklist be read in conjunction with the SPIRIT 2013 Explanation & Elaboration for important clarification on the items. Amendments to the protocol should be tracked and dated. The SPIRIT checklist is copyrighted by the SPIRIT Group under the Creative Commons "Attribution-NonCommercial-NoDerivs 3.0 Unported" license.

For review only

BMJ Open

A person-centered transition program to empower adolescents with congenital heart disease in the transition to adulthood: study protocol for a hybrid randomized controlled trial (STEPSTONES project)

Journal:	*BMJ Open*
Manuscript ID	bmjopen-2016-014593.R2
Article Type:	Protocol
Date Submitted by the Author:	19-Jan-2017
Complete List of Authors:	Acuna Mora, Mariela; University of Gothenburg, Institute of Health and Care Sciences; KU Leuven, Department of Public Health and Primary Care<br>Sparud-Lundin, Carina; University of Gothenburg, Institute of Health and Care Sciences<br>Bratt, Ewa-Lena; University of Gothenburg, Institute of Health and Care Sciences; The Queen Silvia Children's Hospital, Department of Pediatric Cardiology<br>Moons, Philip; KU Leuven, Department of Public Health and Primary Care; University of Gothenborg, Institute of Health and Care Sciences
**Primary Subject Heading**:	Cardiovascular medicine
Secondary Subject Heading:	Research methods, Patient-centred medicine, Paediatrics
Keywords:	Congenital heart disease < CARDIOLOGY, Adolescents, Heart defect, Person centered care, Randomized controlled trial, Transition

SCHOLARONE™ Manuscripts

For peer review only

A person-centered transition program to empower adolescents with congenital heart disease in the transition to adulthood: study protocol for a hybrid randomized controlled trial (STEPSTONES project)

For peer review only

Mariela Acuña Mora MSc, RN[1,2], Carina Sparud-Lundin PhD, RN[1], Ewa-Lena Bratt PhD, RN[1,3], Philip Moons PhD, RN[1,2]

[1] Institute of Health and Care Sciences, University of Gothenburg, Gothenburg, Sweden

[2] KU Leuven Department of Public Health and Primary Care, Leuven, Belgium

[3] Department of Pediatric Cardiology, The Queen Silvia Children's Hospital, Sweden

Mariela Acuña Mora: mariela.acuna.mora@gu.se

Carina Sparud-Lundin: carina.s-lundin@fhs.gu.se

Ewa-Lena Bratt: ewa-lena.bratt@gu.se

Philip Moons: philip.moons@kuleuven.be

Corresponding author:

Philip Moons, PhD, RN, Professor of Healthcare and Nursing Science, Department of Public Health and Primary Care, Kapucijnenvoer 35 Box7001 3000 Leuven-Belgium; Phone: +32 16 373315; philip.moons@kuleuven.be

Word Count: 5241

For peer review only - http://bmjopen.bmj.com/site/about/guidelines.xhtml

ABSTRACT

Introduction

When a young person grows up they evolve from an independent child to an empowered adult. If an individual has a chronic condition this additional burden may hamper adequate development and independence. Transition programs for young persons with chronic disorders aim to provide the necessary skills for self-management and participation in care. However, strong evidence on the effects of these interventions is lacking. Therefore, as part of the STEPSTONES project (Swedish Transition Effects Project Supporting Teenagers with chrONic mEdical conditionS) we propose a trial to assess the effectiveness of a structured, person-centered transition program to empower adolescents with congenital heart disease in the transition to adulthood.

Methods/Design

STEPSTONES will use a hybrid experimental design in which a randomized controlled trial is embedded in a longitudinal, observational study. It will be conducted in four pediatric cardiology centers in Sweden. Two centers will be allocated to the randomized controlled trial group, assigning patients randomly to the intervention group (n=63) or the comparison group (n=63). The other two centers will form the intervention-naïve control group (n=63). The primary outcome is the level of patient empowerment, as measured by the Gothenburg Young Persons Empowerment Scale (GYPES).

Ethics and dissemination

The study has been approved by the Regional Ethical Board of Gothenburg, Sweden. Findings will be reported following the CONSORT statement and disseminated at international conferences and as published papers in peer-reviewed journals.

For peer review only

Trial registration number: NCT02675361

Keywords

Adolescents, congenital heart disease, heart defect, person centered care, randomized controlled trial, transfer, transition.

ARTICLE SUMMARY

Strengths and limitations

- This is a multicenter study that uses a novel study design to test the effectiveness of a person-centered transition program.
- Although several guidelines have recommended the implementation of transition interventions, the evidence of these is still insufficient. The study is expected to fill a gap in current transitional care knowledge.
- Due to the type of intervention, blinding of participants is not possible.
- In order to have a long follow-up, transition interventions are recommended to start before the age of 16 years, which is not feasible in this study.

For peer review only

For peer review only - http://bmjopen.bmj.com/site/about/guidelines.xhtml

BACKGROUND

In developed countries, 10-40% of the adolescent population has a chronic disease with childhood onset.[1] As a result of improvements in diagnostic techniques and treatments, survival has increased, and 90% of them now reach adulthood.[2] Growing up with a chronic condition poses an extra burden on the individual, over and above the challenges typically related to adolescence. Management of the transition from childhood to adulthood and the shift from pediatric to adult care requires special attention from healthcare professionals. The movement to adult care is known as transfer, defined as "*an event or series of events through which adolescents and young adults with chronic physical and medical conditions move their care from a pediatric to an adult health care environment*".[3] This transfer should be preceded by a preparatory transitional phase. In healthcare, transition can be defined as the *"process by which adolescents and young adults with chronic childhood illnesses are prepared to take charge of their lives and their health in adulthood"*.[3] To improve and facilitate this process, transition programs have been developed.[4] A well designed transition program is individually-tailored, flexible and age and developmentally appropriate.[5] The goal of transition programs is to support the person's adjustment to a new care culture, to provide the necessary tools for self-care and to help satisfy medical, psychosocial and educational needs.[6] Evidence on the effects of transition programs is scant, the level of existing evidence is low and the effectiveness of these interventions has yet to be determined.[7] This is due to the limited number of effectiveness studies; methodological issues (e.g. contamination of control groups has not been accounted for in prior studies) and a short follow-up period of 12 months maximum.[7]

Patient empowerment is an asset that relates to the adolescents' self-management and participation in care[8]. Empowered adolescents can interact better with the adult healthcare system, are more independent and more actively involved in decisions regarding their

For peer review only - http://bmjopen.bmj.com/site/about/guidelines.xhtml

health.[9,10] However, patient empowerment has not been a formal target in transition programs so far. To increase the level of empowerment, a person-centered care approach is important.[11] This perspective allows for a partnership between the adolescent and healthcare providers in which the adolescent's views guide the intervention.[12] Through this approach it is possible to share helpful resources and information with the adolescents and give them the opportunity to assume an active role in the decision-making process. [11]

Congenital heart disease (CHD) is a typical example of a life-course disease and can therefore be considered a chronic condition. CHD has a prevalence of 9.1 per 1000 newborns and represents 28% of all congenital defects.[13] Around 90% of children born with CHD reach adulthood,[2] and approximately 6.2 in 1000 adults live with this condition.[14] Although several centers have established a transition program for individuals with CHD,[15] only one study on the effectiveness of such a program has been undertaken so far. That study was conducted in Canada and used a quasi-experimental design to test the effects of a one-hour intervention led by a nurse.[16] Significant improvements in the level of knowledge and self-management among patients were observed.[16] In addition, a comparative and a longitudinal study in Belgium confirmed the effectiveness of patient education.[17, 18]

To address the gaps in evidence on the effectiveness of transition programs, we established the STEPSTONES project (Swedish Transition Effects Project Supporting Teenagers with chrONic mEdical conditionS). This project aims at developing and evaluating the effectiveness of transition programs for young persons with chronic childhood-onset conditions. Although the project will encompass different chronic conditions, it will first study adolescents with CHD.

Objective and hypothesis

For peer review only

This article describes the protocol for a study to test the effectiveness of a transition program to empower adolescents with congenital heart disease in the transition to adulthood and the transfer to adult care. The main hypothesis is that adolescents with CHD who receive a structured, person-centered transition program over a 2-year period have a higher patient empowerment score than adolescents receiving usual care.

This study is presented in accordance with the guidelines issued by SPIRIT for reporting of study protocols.[19]

METHODS/DESIGN

Study design and setting

A hybrid experimental design will be used, in which a randomized controlled trial (RCT) is embedded in a longitudinal, observational study. This will result in a 3-arm design (see Figure 1). The study will be conducted in four CHD-centers in Sweden. Two of the centers are allocated to the RCT, where participants are randomly assigned to either the intervention group or the comparison group. The other two centers are designated as the control group and represent intervention-naïve centers.

This type of design allows for the investigation of the effectiveness of the transition program while also taking into account potential contamination of the comparison group in the intervention centers.

Sample size estimation

For peer review only - http://bmjopen.bmj.com/site/about/guidelines.xhtml

Sample size calculation is based on the primary outcome of patient empowerment. The target is an improved patient empowerment score of 5.25 points on a scale from 15-75 (i.e. 0.5 standard deviation). For two-sided tests with alpha=0.05 and power=80%, 63 patients are needed in each arm of the RCT. In order to compensate for a potential 10% dropout rate, we will recruit 70 patients in each arm of the RCT. An additional 70 patients will be recruited in the centers of the control group. Among the four centers a total of 210 patients will be enrolled (See Figure 1).

Participants and recruitment

Eligible participants are 1) adolescents with CHD, defined as "*a gross structural abnormality of the heart or the intrathoracic great vessels that is actually or potentially of functional significance*";[20] 2) age 16 years; 3) Swedish speaking; and 4) literate.

Patients are excluded if they do not have the physical or cognitive capacity to complete the questionnaires, have acquired heart conditions or heart transplantation. Parents of the adolescents will also be asked to participate. All eligible participants are recruited by a transition coordinator (TC) or a data collection officer (DCO).

Randomization

Using a web-based system, a stratified block randomization with random variable block size will assign patients of the RCT centers to the intervention group or the comparison group (1:1).[21] Block randomization ensures that the transition coordinators (TCs) in the two centers have a relatively continuous exposure to the intervention over time, which allows them to keep their skills up-to-date and to ensure the fidelity of the intervention. The fact that the

For peer review only - http://bmjopen.bmj.com/site/about/guidelines.xhtml

block sizes vary randomly minimizes the possibility that the TCs could predict group assignments.

Stratification is made by center and disease complexity. The stratification for center ensures that the two centers have an equal number of intervention patients. Stratification by disease complexity (mild; moderate; complex)[22] is important to avoid overrepresentation of that people with, for example, complex CHD, in one or the other group due to failing randomization.

Overall this approach decreases the within centers variability and reduces the risk for bias and confounding.[21] The TCs are responsible for accessing the randomization system in order to determine patient allocation.

Intervention

Transition program (intervention group)

This study implements a multicomponent intervention, adapted from a brief transition program developed by Hilderson.[23] Adaptation of the program followed the intervention mapping process.[24] Intervention mapping is a thorough method used to build comprehensive programs for health promotion and consists of 6 consecutive steps: 1) assessment of the needs of the target population; 2) defining program objectives by specifying performance and change objectives; 3) determining theory-based intervention methods and practical strategies; 4) designing and producing the intervention program; 5) adoption and implementation of the intervention; and 6) developing and executing a plan for evaluation.[24] Applying the intervention mapping method in this study ensures that the intervention is well-grounded and

For peer review only - http://bmjopen.bmj.com/site/about/guidelines.xhtml

includes the necessary components to target the program objectives. A detailed description of each of the steps will be given in a related article.

The transition program includes 8 key components: a) a transition coordinator (TC); b) a written person-centered transition plan; c) provision of information and education about the condition, treatment and health behaviors; d) availability by telephone and email; e) information about and contact with the GUCH clinic (Grown-Up Congenital Heart Disease); f) guidance of parents; g) meeting with peers; and h) the actual transfer to the GUCH clinic.

These components are implemented in five steps: 1) a first patient visit with the TC; 2) second outpatient visit with TC; 3) information day for adolescents and their parents; 4) third outpatient visit with TC; and 5) actual transfer to the GUCH clinic. The TCs are specialized nurses at the outpatient clinic of pediatric cardiology that have received specific training in performing this intervention.

Throughout the intervention the TCs employ a person-centered care (PCC) approach. PCC implies that participants are active agents in their care and decision-making process.[12] Ekman proposes that PCC can be implemented in three stages.[12] First, initiating the partnership by allowing the patients to tell their story (patient narratives). This allows for the patients' views to be at the center of care. Second, developing the partnership through constant communication between the patient and healthcare providers. This is accomplished by sharing experiences and building a common understanding of the care plan. Third, safeguarding the partnership by documenting the decisions made and the preferences of the patients.

The content of the visits is individualized, age and developmentally adapted and it is documented in the individualized transfer plan. The TCs together with the adolescent are in

For peer review only - http://bmjopen.bmj.com/site/about/guidelines.xhtml

charge of determining which topics are important to discuss, in establishing goals related to patient empowerment and in assessing the need for referral to other services.

Usual care (intervention, control and comparison group)

All adolescents who participate in the study receive usual care, irrespective of whether in the intervention, comparison or control group. Usual care includes visits to an outpatient pediatric cardiology clinic where they meet a nurse and a pediatric cardiologist. Disease complexity determines the frequency of the visits. None of the participating centers have a TC as a part of usual care and there is no documentation of transition plans. It is common practice in Sweden that patients are transferred to adult care around the age of 18 years.

Since usual care can vary across clinics and over time, an assessment of transition-related practices will be done in all the centers at the beginning of the intervention and at the end of the study. This assessment will be comprised of three elements: 1) a structured survey called CHAT (Congenital Heart Adolescent and Transition) (on file); 2) interviews with healthcare professionals who are involved in the care for adolescents with CHD in the participating centers; and 3) observations of the routine during the clinic visits.

Blinding

Due to the nature of the intervention and the study design, it is not possible to blind the participants, TCs or data collection officers (DCOs). However, the TCs and DCOs in charge of carrying out the intervention and data collection are not involved in the preparation of the intervention design or in the statistical analyses.

For peer review only - http://bmjopen.bmj.com/site/about/guidelines.xhtml

To avoid cross contamination between the comparison and the intervention group within the RCT centers, TCs are not involved in the delivery of care of the comparison group and other healthcare professionals in the participating centers will not be informed of the intervention components. DCOs in the control group do not participate in discussions of the intervention.

Intervention fidelity

To assure that the transition program is implemented according to the developed intervention, the TCs are selected based on their experience of working with children and adolescents with CHD. In preparation for this study, they received a specifically designed 4-days training program, addressing adolescents' health and development; communicating with and interviewing young persons; patient education; person-centered care; and the detection of and screening for risk behaviors. Training is given by experts in the aforementioned fields and features different pedagogical approaches, e.g. group discussions, case assessments, and role play. In addition, the TCs were asked to read two textbooks on adolescent health and person-centered care.

Standard Operating Procedures (SOPs) for each group of the study were developed in order to ensure consistency throughout the study. These SOPs included: ethical approval; study preparation; study administration; participants recruitment and survey completion; intervention characteristics; usual care; data storage, entry and security; study progress; and publication policy.

Intervention fidelity is monitored as part of the process evaluation. The Medical Research Council (MRC) guidance on process evaluation of complex interventions is applied,[25] and both quantitative and qualitative methods will be used. In the quantitative

For peer review only - http://bmjopen.bmj.com/site/about/guidelines.xhtml

assessment we investigate the acceptability, adherence/fidelity and attrition, as well as potential safety issues. These data are collected using three assessment forms (enrolment/follow-up form, adverse events report and intervention implementation form) filled out by the TCs during the intervention period. The qualitative assessment will obtain information about the experiences of and implementation by the TCs and will provide insights into the mechanisms of impact based on the experiences of patients and their parents. The process evaluation will be undertaken as a separate study conducted in parallel with this effectiveness study.

Follow-up period and data collection

During the 2-year follow-up period, outcome measurements are assessed on three different occasions: T0, baseline when the participants are 16 years old; T1, midterm when the participants are 17 years; and T2, final data collection when the participants have been transferred to adult care and are approximately 18.5-19 years old (See Figure 1).

Participants from the intervention group will be mailed the set of questionnaires one month before the outpatient clinic visit. If the questionnaires have not been received by the time of the visit, they will have the opportunity to complete them while waiting for their appointment. Patients from the control and comparison groups may not have a scheduled outpatient visit. Therefore, data collection in these groups is completely undertaken by post when the participants are 16 (T0), 17 (T1) and 18.5 years old (T2).

To minimize non-response in the comparison and control groups a modified Dillman procedure will be used.[26] Two weeks after the documents are sent, non-responders will receive a first personalized reminder in the form of a text message. If after four weeks the questionnaires have not been received a reminder letter and a new set of questionnaires is

For peer review only - http://bmjopen.bmj.com/site/about/guidelines.xhtml

sent. After six weeks, persistent non-responders are contacted by telephone and asked whether they received the questionnaires, if the mailing address is correct, if they want a new set of questionnaires and if they want to continue participating in the study. After 8 weeks a last reminder is sent for those who agree to participate in the study during the telephone call.

Outcome measurements

A background information questionnaire is included, comprising sociodemographic variables such as sex, age, educational level, size of community, place of birth, number of siblings, birth order, living situation, other health conditions and medication (obtained from self-report and medical file).

Standardized questionnaires are used to assess primary and secondary outcomes. Table 1 presents an overview of the characteristics and psychometric properties of the questionnaires used.

For peer review only

Table 1: Psychometric properties of the questionnaires used in STEPSTONES-ConHD							
**Variable**	**Subject**	**Measurements**	**Items**	**Validity**	**Reliability**	**Responsiveness**	**Interpretation**
Primary Outcome							
Patient empowerment	AD	Gothenburg Young Persons Empowerment Scale (GYPES)	15	Construct validity supported an adequate model fit of a five factor solution (*df*: 80; χ2: 154-948, p<0.0001; CFI: 0.916; RMSEA: 0.068; SRMR: 0.069) (data on file)<br>Face validity confirmed in young persons with chronic conditions (data on file)	Internal consistency confirmed in young persons with CHD, α=0.819 (data on file)	Floor scores: 0%; Ceiling scores: 1.5% (data on file)	Score from 15-75. Higher score reflecting a higher level of patient empowerment
Secondary Outcomes							
Transition readiness	AD	Readiness for Transition Questionnaire (RTQ) adolescent version	26	Validity based on relationships with other variables confirmed in young persons with kidney transplant, [27] p.91-92	Internal consistency confirmed in young persons with kidney transplant; α values over 0.70,[27]	NR	Scores from 10-40. Higher scores denote increased adolescent or caregiver responsibility
	PA	Readiness for Transition Questionnaire (RTQ) parent version		Construct validity reported for parents of young persons with kidney transplant, [27] p.91-92	Reliability reported for parents of young persons with kidney transplant; α values over 0.70, [27] p.90	NR	

For peer review only - http://bmjopen.bmj.com/site/about/guidelines.xhtml

Knowledge on ConHD	AD	Knowledge Scale for Adults with Congenitally Malformed Hearts (KnoCoMH)	19	Validity was confirmed in adults with CHD in relation to discrimination ability, the relationship of the items and the construct of interest, [28] p.232-234<br><br>KnoCoMH is based on the Leuven Knowledge Scale which has been validated in young persons with CHD, [29] p.79-80	Internal consistency and test-retest reliability confirmed,[28] p.232-234	NR	Scores are calculated by dichotomizing the answers (correct/incorrect) for each domain
Health behaviors	AD	Health Behavior Scale-Congenital Heart Disease (HBS-CHD)	15*	Item content validity, scale content validity index and validity based on relationships to other variables confirmed in adolescents with CHD, [30] p.548	Stability not confirmed.[29]	Confirmed in adolescents with CHD by Guyatt's Responsiveness Index, [30] p.551	Substance use, dental hygiene and total health risk score from 0-100. Physical exercise score from 0-∞
Patient-reported health		Pediatric Quality of Life Inventory 4.0 (PedsQL) generic module	23	Convergent validity, measurement invariance and factor structure confirmed in pediatric populations,<br><br>[31] p.336-337<br><br>[32] p.1507-1510	Test-retest reliability, and internal consistency show α values over 0.70 in pediatric populations, [32] p.1507<br><br>[31] p.333-335	Minimal clinically important differences reported for young people with CHD was 6.0 for the total score,[33]	Scores from 0 to 100. Higher scores indicate a better perceived health status
	AD	Pediatric Quality of Life Inventory 4.0 (PedsQL) cardiac module	27	Convergent validity was confirmed, [34] p.215	Internal consistency for majority of scales exceeded α values of 0.70, [34] p.214	Minimal clinically important differences reported for young people with CHD ranged from 7.6	Scores from 0 to 100. Higher scores indicate lower problems

For peer review only - http://bmjopen.bmj.com/site/about/guidelines.xhtml

						for symptoms to 12.6 for communication, [33]	
		EQ-5D-3L	5	Convergent and discriminative validity confirmed in cardiovascular patients, [35] p.6	Test-retest reliability confirmed in cardiovascular patients, [35] p.6	Confirmed in cardiovascular patients, [35] p.6	Scores from 1 (no problems) to 3 (extreme problems) on five dimensions.<br><br>Scores from 0 (worst imaginable health state) to 100 (best imaginable health state)
Quality of life	AD	Linear analog scale (LAS)	1	Validity based on relationship with Satisfaction with Life Scale found to be highly correlated (ρ=0.52). Test content confirmed in adults with CHD (100% of patients understood the wording and format), [36]<br><br>Validity has been evaluated in adolescents with CHD [37, 38]	Test-retest reliability confirmed in adults with CHD (ICC=0.65), [36]	Confirmed in adults with CHD (Floor scores= 0%, ceiling scores=2.7%), [36]	Score is from 100 (best imaginable health status) to 0 (worst imaginable health status)
Healthcare utilization	AD	Healthcare utilization questionnaire	11	NA	NA	NA	NA
Parental uncertainty towards transfer to adult care	PA	Linear analog scale (LAS)	1	Face validity confirmed in parents of young persons with chronic conditions (data on file).<br><br>Test content confirmed in	NR	Floor scores: 7.8%; Ceiling scores: 4.8% (data on file)	Score is from 0 (very uncertain about the transfer to adult care) to 100 (not uncertain

For peer review only - http://bmjopen.bmj.com/site/about/guidelines.xhtml

				parents of young persons with CHD (100% of patients understood the wording and format).			about the transfer of care)
AD: adolescents. PA: parents. NA: not applicable. NR: not reported. CFI: comparative fit index. RMSEA: root mean square error of approximation. SRMR: standardized root mean square residual. $\chi 2$: chi-square test of model fit.							

For peer review only

Primary outcome measures

The primary outcome is patient empowerment, defined as "*an enabling process or outcome arising from communication with the healthcare professional and a mutual sharing of resources over information relating to illness, which enhances the patient's feelings of control, self-efficacy, coping abilities and ability to achieve change over their condition.*[9] Empowerment includes five dimensions: 1) identity; 2) knowledge and understanding; 3) personal control; 4) decision making; and 5) enabling others.[9]

Since no questionnaire to measure the level of empowerment in young persons with chronic conditions exists, we developed the Gothenburg Young Persons Empowerment Scale-Congenital Heart Disease module (GYPES-CHD). The scale has 15 items, 3 items for each of the aforementioned dimensions. Each item is answered on a five-point Likert scale: strongly disagree; disagree; neither agree or disagree; agree; and strongly agree. A total score is calculated by summing the item scores. The total score ranges from 15-75 points, with a higher score reflecting a higher level of patient empowerment.

Face validity was confirmed after cognitive interviews with 6 young persons with CHD. The psychometric properties of this scale were examined in a cross-sectional study in 205 young persons with CHD. Internal consistency of the items was confirmed with a Cronbach's alpha of 0.821. No floor or ceiling effect in scoring was observed. A complete description of the psychometric evaluation of GYPES will be provided in another article.

Secondary outcomes measures

We will measure six secondary outcomes in the adolescents: transition readiness, health behaviors, knowledge about CHD, healthcare utilization, quality of life and patient-reported health. In the parents, we will measure transition readiness (proxy) and parental uncertainty toward transition.

For peer review only - http://bmjopen.bmj.com/site/about/guidelines.xhtml

Transition readiness

Transition readiness is defined as the "*adolescent's readiness to assume complete responsibility for their healthcare and their readiness to transfer to adult medical care*".[27] Transition readiness is evaluated using the Readiness for Transition Questionnaire (RTQ), adolescent and parent versions. These questionnaires examine two aspects. First the overall transition readiness is assessed, using two items ranging from 1-4. The sum of these two items results in a score ranging from 2-8. Second, the frequency of adolescent responsibility and parental involvement are reported in ten different health behaviors, on a five-point Likert. Each variable results in a total score ranging from 10 to 40, higher scores indicate higher adolescent responsibility or parental involvement, respectively.

Knowledge about CHD

Adolescents' knowledge about their CHD is assessed with the Knowledge Scale for adults with Congenital Malformed Hearts (KnoCoMH). [28] This instrument evaluates the level of knowledge within four domains: disease and its treatment; complications prevention; physical activity; and reproductive issues.[28] The questionnaire includes 19 items and scores are calculated by dichotomizing answers as correct or incorrect. Access to the medical files is needed in order to confirm the answers in some of the items. For participants where medical treatment or endocarditis prophylaxis is not recommended, the domains are excluded.

Health behaviors

Health behaviors are measured with the Health Behavior Scale-CHD. Health behaviors are *"activities a person undertakes to maintain or improve health and prevent diseases"*.[30] This scale assesses alcohol consumption, tobacco use, dental care and physical activity. The scale has 15 items that help to calculate three summary risk scores: substance

For peer review only - http://bmjopen.bmj.com/site/about/guidelines.xhtml

use (score 0-100), dental hygiene (score 0-100) and total health risk score (score 0-100). Higher risk scores represent unhealthier behaviors.

Patient-reported health

Patient-reported health is assessed by the generic and cardiac modules of the Pediatric Quality of Life inventory 4.0 (PedsQL 4.0), as well as the EuroQoL 5 dimensions 3 levels (EQ-5D-3L). The PedsQL 4.0 provides information on health status in relation to psychosocial and physical health.[32] The generic module consists of 23 items that cover four dimensions: physical; emotional; social functioning; and school functioning. Items are answered by using a five-point Likert scale: never, almost never, sometimes, often, and always. For each dimension, a scale score can be calculated, which ranges from 0 to 100. Further, a physical health summary score, a psychosocial health summary score, and a total score can be calculated.

The cardiac module contains 27 items that cover six dimensions: symptoms; treatment; perceived physical appearance; treatment anxiety; cognitive problems; and communication. Each item is answered using a 5-point Likert scale (never, almost never, sometimes, often and always). As in the generic version, scores are transformed on a scale from 0 to 100, and then a mean subscale core is calculated.[34]

The EQ-5D-3L comprises a descriptive system and a visual analog scale (VAS). The descriptive system has 5 dimensions (mobility, self-care, usual activities, pain/discomfort and anxiety/depression) with 3 response categories per dimension.[39] The participants are asked to indicate which statement is most suitable in relation to their health status. The VAS measures the participants' self-rated health on a scale with the endpoints "best imaginable health possible" (=100) and "worst imaginable health state (=0)". The descriptive system allows for the determination of a 5 digit health state that combines the level of perceived problems in

For peer review only - http://bmjopen.bmj.com/site/about/guidelines.xhtml

each dimension (level 1: indicating no problem, level 2: indicating some problems and level 3: indicating extreme problems). Information obtained from the descriptive system can be converted to a single summary index by assigning values to each level of every dimension.

Quality of life

Quality of life is defined as "*the overall degree of life satisfaction that is positively or negatively influenced by individuals' perception of certain aspects of life important to them, including matters both related and unrelated to health"*.[40] This variable is measured using a 10 cm linear analog scale (LAS), [36] where the participants indicate in the scale how they judge their current quality of life. The scale looks similar to the VAS of the EQ-5D, but has the endpoints "best imaginable quality of life" (=100) and "worst imaginable quality of life" (=0).

Healthcare utilization

Healthcare utilization within the preceding six months is measured in relation to their ConHD and other possible conditions. The measurement consists of six dimensions: hospital stay; healthcare contact; specialized care; emergency care; school absence; and follow-up unit after transfer to adult care. This assessment was developed to be used in the APPROACH-IS project.[41] After the transfer to adult care, we will assess the proportion of patients that are in follow-up in a GUCH unit, both in the intervention as control arms.

Parental uncertainty

Parental uncertainty is understood as the degree of certainty/uncertainty the parents have in relation to their children being transferred to adult care. This outcome is measured through LAS with the endpoints "extremely uncertain" (=100) and "not uncertain at all" (=0). This LAS has been developed for the purpose of the present study.

For peer review only - http://bmjopen.bmj.com/site/about/guidelines.xhtml

In addition to the primary and secondary endpoints, we will calculate a composite endpoint. We will do so because the transition program is a complex intervention that may have varying effects on different outcomes. Prior studies on the impact of transition programs showed that the intervention has impacted on transition readiness,[16] patient knowledge,[16] quality of life,[42] and psychosocial health.[42] Therefore, each patient will be categorized as improved or not. Patients will be classified as improved if they have an improvement in empowerment, transition readiness, knowledge, quality of life, or psychosocial health of 0.5 standard deviations compared to their baseline measurement.

Data management

Data are entered into a web-based system by the TC and the DCO. Those with access to the system are the TC, DCO, project coordinator and principal investigator, and each have a personal login. All data are checked for completeness and the quality of the data is monitored at least twice a year by the members of the research group. This process is done by verifying records with the raw data and checking frequency distributions to identify responses outside of the given possibilities.

Statistical analyses plan

The primary analyses will be done following the intention to treat principle which helps to avoid biases in the comparisons between groups. The primary endpoint (level of empowerment) will be analyzed using Fishers non-parametric permutation test unadjusted between the two groups. Complementary between-group analyses will be made using

For peer review only - http://bmjopen.bmj.com/site/about/guidelines.xhtml

ANCOVA adjusted for baseline variables that differ significantly between groups and for each center.

All analyses will be predefined in a comprehensive statistical analysis plan before data lock. The level of significance will be at *p value* equal to or less than 0.05. Two-sided tests will be used.

Trial duration

Patient recruitment began July 2016 and is planned to finish in December 2017. Once recruited, patients are followed-up until they are transferred to GUCH. Data collection is expected to be finished by December 2019. After the data set is locked, analysis and dissemination of results will begin.

Ethical considerations

The study was approved by the Regional Ethical Board of Gothenburg, Sweden (No.931-15). Before inclusion in the study, the TCs and DCOs will seek written informed consent from the adolescents and their parents after they have received written and oral information about the study. Participants have the right to withdraw at any point throughout the study. The principles established in the Declaration of Helsinki will be followed during the entire duration of the study.[43]

To ensure confidentiality personal data is coded. Participant and study-related information is stored in locked cabinets and digital documents are password-protected.

For peer review only - http://bmjopen.bmj.com/site/about/guidelines.xhtml

Follow-up on adverse events

All adverse events (AEs) must be reported to the principal investigator and project coordinator within a week. In case of a serious AE, the report must be made immediately. Appropriate measures will be undertaken in every AE in order to ensure patient safety.

Dissemination

The results of this study will be submitted to peer reviewed journals for publication following the CONSORT statement.[44] Abstracts for poster or oral presentations will be submitted to international conferences. Authorship is granted to a collaborator from every participating center and members of the research group fulfilling the criteria of authorship [45, 46]. If possible, we will include "on behalf of STEPSTONES-CHD" in the list of authors. After completion of the study, the data set will be available upon formal request.

DISCUSSION

Previous studies have tried to determine the effectiveness of transition programs. However, due to several methodological limitations, the true effect of these interventions is still uncertain.[7] Therefore, we will conduct a study that assesses the effectiveness of a structured, person-centered transition program to empower adolescents with CHD in the transition to adulthood. Through the implementation of the proposed transition program, we expect an increase in the level of empowerment. A high level of empowerment can help the adolescent navigate through the adult care system, participate in care planning and increase self-management and decision-making skills.[8]

For peer review only - http://bmjopen.bmj.com/site/about/guidelines.xhtml

The intervention in this trial was designed and initially evaluated by Hilderson,[23, 42] in Belgium. Therefore, it was important to determine if the intervention components were suitable in the Swedish setting. To accomplish this, five preparatory studies were done (four qualitative, one quantitative study). The qualitative studies included individual and focus group interviews with young persons with CHD and their parents.[47, 48] These studies provided an overview of the current transition practices in Sweden and the needs of this population before the transfer to adult care. The quantitative study was a cross-sectional study including 205 young persons with CHD, and served as a pilot-test for our primary outcome measurement. Furthermore, the intervention was discussed with a panel of international experts with experience in transition research in adolescents with chronic conditions and patient representatives.

The preparatory studies and the discussion with the panel of international experts improved the quality, feasibility and acceptability of the intervention. This interdisciplinary and comprehensive approach increases the chance of producing a high-quality intervention with positive results.[49]

The present study has several strengths, because we explicitly take limitations of prior studies into account. First, we employ a RCT which is the gold standard for the assessment of the effectiveness of interventions and provides the highest level of evidence. The stratified block randomization that we undertake ensures that the intervention and comparison groups are balanced and that potential confounding factors, such as complexity of the disease or center characteristics, are equally distributed and therefore minimize the risk of biasing the results.

Second, the proposed hybrid experimental design allows us to account for potential contamination of the control group. Indeed, the 3 arms allow performance of direct

For peer review only - http://bmjopen.bmj.com/site/about/guidelines.xhtml

comparison between the intervention and comparison group while enabling a comparison of findings with those obtained in intervention-naive centers.

Third, patient empowerment is used as the primary outcome. Empowerment is argued to be important for the development and application of skills to promote behavioral changes in the adolescents.[10] Admittedly, empowerment can be seen both as a process and an outcome.[10] However, being an intermediate outcome, empowerment can be argued to be proximal to the intervention whereas continuing cardiac care, reduced morbidity or mortality are probably the ultimate, but more distal outcomes.

Fourth, the PCC approach that is used throughout the transition program is an important asset in the process of empowering patients.[11] Providing transitional care from a person-centered perspective is novel and has not been evaluated before in this age group.

Fifth, we follow-up the patients for more than 2 years. Doing so, we substantially expand the follow-up period of 12 months as used in prior studies.[7]

Sixth, since usual care may vary across centers and over time, we carry out a formal usual care assessment. Such a usual care assessment is important to draw firm conclusions and yet this is seldom performed in clinical trials.[50]

Irrespective of these strengths, we anticipate some limitations as well. First, we are not able to blind the participants or the healthcare team that will perform the intervention. To minimize a potential impact, neither the therapeutic team nor the TCs participate in the data analysis. Second, there is a possibility of a relatively long period of recruitment for participants which can require extending the trial period and delaying data analysis. Third, transition interventions have been advised to start early on, e.g. between 10-12 years of age, and have a long follow-up.[51] Furthermore, a long follow-up allows measuring continuity of care in this population and provide insight on the effects of the transition program over this

For peer review only - http://bmjopen.bmj.com/site/about/guidelines.xhtml

parameter. Although we recognize the importance of starting early, it would have jeopardized the feasibility of the study, because it would have taken almost a decade to finish recruitment and follow-up until the age of 18 years. Nonetheless, it could be possible to later on, assess loss of follow-up in a separate study

Conclusion

This article described the study protocol for a study to assess the effectiveness of a structured, person-centered transition program for young persons with CHD. This study accounts for limitations of previous research and addresses an existing gap in the evidence of transitional care. It is hoped the findings will provide strong evidence of the effectiveness of this intervention in young people with CHD. If successful, this transition program could potentially be implemented as part of routine care provided to young persons with CHD, and transfer to other chronic conditions can be explored.

Trial status

Status of the trial at the time of submission: patient recruitment

List of abbreviations

CHD: congenital heart disease

DCO: data collection officer

GYPES: Gothenburg Young Persons Empowerment Scale

For peer review only - http://bmjopen.bmj.com/site/about/guidelines.xhtml

GYPES-CHD: Gothenburg Young Persons Empowerment Scale-Congenital Heart Disease module

GUCH: grown-up congenital heart disease unit

MRC: Medical Research Council

RCT: randomized controlled trial

STEPTONES: Swedish Transition Effects Project Supporting Teenagers with chrONic mEdical conditionS

TC: transition coordinator

Competing interests

There are no conflicts of interests.

Funding

This study is supported by research grants from the Swedish Heart-Lung Foundation; Swedish Research Council for Health, Working Life and Welfare; Swedish Children Heart Association; Swedish Research Council; and the Institute of Health and Care Sciences of the University of Gothenburg.

Author's contributions

For peer review only - http://bmjopen.bmj.com/site/about/guidelines.xhtml

For peer review only

MAM, CSL, ELB and PM conceived the study, were responsible for the design of the study protocol and drafted the manuscript. All authors critically revised and approved the manuscript.

Author's information

MAM is a PhD student at the Institute of Health and Care Sciences, University of Gothenburg, Sweden and at the Academic Center for Nursing and Midwifery, Department of Public Health and Primary Care, KU Leuven, Belgium. CSL is senior lecturer at the Institute of Health and Care Sciences, University of Gothenburg, Sweden. ELB is senior lecturer at the Institute of Health and Care Sciences, University of Gothenburg, Sweden and a pediatric nurse in Queen Silvia Children's Hospital. PM is Professor of Healthcare and Nursing Science and Program Director at the KU Leuven Department of Public Health and Primary Care, Belgium and Visiting Professor at the Institute of Health and Care Sciences, University of Gothenburg, Sweden.

Acknowledgements

We would like to acknowledge the collaboration from the members of the panel of international experts and of the Advisory Board.

References

1. Kennedy A, Sloman F, Douglass JA, et al. Young people with chronic illness: the approach to transition. Intern Med J 2007;**37**(8):555-60.

For peer review only - http://bmjopen.bmj.com/site/about/guidelines.xhtml

2. Moons P, Bovijn L, Budts W, et al. Temporal trends in survival to adulthood among patients born with congenital heart disease from 1970 to 1992 in Belgium. Circulation 2010;**122**(22):2264-72.

3. Meadows AK, Bosco V, Tong E, et al. Transition and transfer from pediatric to adult care of young adults with complex congenital heart disease. Curr Cardiol Rep 2009;**11**(4):291-97.

4. American Academy of Pediatrics. A consensus statement on health care transitions for young adults with special health care needs. Pediatrics 2002;**110**(6 Pt 2):1304-6.

5. Warnes CA, Williams RG, Bashore TM, et al. ACC/AHA 2008 Guidelines for the Management of Adults With Congenital Heart Disease: A Report of the American College of Cardiology/American Heart Association Task Force on Practice Guidelines (Writing Committee to Develop Guidelines on the Management of Adults With Congenital Heart Disease): Developed in Collaboration With the American Society of Echocardiography, Heart Rhythm Society, International Society for Adult Congenital Heart Disease, Society for Cardiovascular Angiography and Interventions, and Society of Thoracic Surgeons. Circulation 2008;**118**(23):E714-E833.

6. Crowley R, Wolfe I, Lock K, et al. Improving the transition between paediatric and adult healthcare: a systematic review. Arch Dis Child 2011;**96**(6):548-53.

7. Campbell F, Biggs K, Aldiss SK, et al. Transition of care for adolescents from paediatric services to adult health services. Cochrane Database Syst Rev 2016;**4**:CD009794.

8. Bravo P, Edwards A, Barr PJ, et al. Conceptualising patient empowerment: a mixed methods study. BMC Health Serv Res 2015;**15**:252.

9. Small N, Bower P, Chew-Graham CA, et al. Patient empowerment in long-term conditions: development and preliminary testing of a new measure. BMC Health Serv Res 2013;**13**:263.

10. Anderson RM, Funnell MM. Patient empowerment: Myths and misconceptions. Patient Educ Couns 2010;**79**(3):277-82.

11. Holmstrom I, Roing M. The relation between patient-centeredness and patient empowerment: a discussion on concepts. Patient Educ Couns 2010;**79**(2):167-72.

12. Ekman I, Swedberg K, Taft C, et al. Person-centered care--ready for prime time. Eur J Cardiovasc Nurs 2011;**10**(4):248-51.

For peer review only

For peer review only - http://bmjopen.bmj.com/site/about/guidelines.xhtml

13. Dolk H, Loane M, Garne E, et al. Congenital heart defects in Europe: prevalence and perinatal mortality, 2000 to 2005. Circulation 2011;**123**(8):841-9.

14. Marelli AJ, Ionescu-Ittu R, Mackie AS, et al. Lifetime prevalence of congenital heart disease in the general population from 2000 to 2010. Circulation 2014;**130**(9):749-56.

15. Hilderson D, Saidi AS, Van Deyk K, et al. Attitude toward and current practice of transfer and transition of adolescents with congenital heart disease in the United States of America and Europe. Pediatr Cardiol 2009;**30**(6):786-93.

16. Mackie AS, Islam S, Magill-Evans J, et al. Healthcare transition for youth with heart disease: a clinical trial. Heart 2014;**100**(14):1113-8.

17. Goossens E, Fieuws S, Van Deyk K, et al. Effectiveness of structured education on knowledge and health behaviors in patients with congenital heart disease. J Pediatr 2015;**166**(6):1370-6 e1.

18. Goossens E, Van Deyk K, Zupancic N, et al. Effectiveness of structured patient education on the knowledge level of adolescents and adults with congenital heart disease. Eur J Cardiovasc Nurs 2014;**13**(1):63-70.

19. Chan AW, Tetzlaff JM, Altman DG, et al. SPIRIT 2013 statement: defining standard protocol items for clinical trials. Ann Intern Med 2013;**158**(3):200-7.

20. Mitchell SC, Korones SB, Berendes HW. Congenital heart disease in 56,109 births. Incidence and natural history. Circulation 1971;**43**(3):323-32.

21. Efird J. Blocked randomization with randomly selected block sizes. Int J Environ Res Public Health 2011;**8**(1):15-20.

22. Warnes CA, Liberthson R, Danielson GK, et al. Task force 1: the changing profile of congenital heart disease in adult life. J Am Coll Cardiol 2001;**37**(5):1170-5.

23. Hilderson D, Westhovens R, Wouters C, et al. Rationale, design and baseline data of a mixed methods study examining the clinical impact of a brief transition programme for young people with juvenile idiopathic arthritis: the DON'T RETARD project. BMJ Open 2013;**3**(12):e003591.

24. Bartholomew LK, Markham CM, Ruiter R, et al. *Planning health promotion programs : an intervention mapping approach*. 4th ed. San Francisco: Jossey-Bass, 2016.

For peer review only

For peer review only - http://bmjopen.bmj.com/site/about/guidelines.xhtml

25. Moore GF, Audrey S, Barker M, et al. Process evaluation of complex interventions: Medical Research Council guidance. BMJ 2015;**350**:h1258.

26. Dillman D. Mail and other self-administered questionnaires. In: Rossi PH, Wright JD, Anderson AB, eds. Handbook of survey research. New York: Academic Press, 1983:pp. 359–77.

27. Gilleland J, Amaral S, Mee L, et al. Getting ready to leave: transition readiness in adolescent kidney transplant recipients. J Pediatr Psychol 2012;**37**(1):85-96.

28. Ronning H, Arestedt K, Erik Nielsen N, et al. Development and psychometric evaluation of the knowledge scale for adults with congenitally malformed hearts. J Cardiovasc Nurs 2013;**28**(3):228-37.

29. Yang HL, Chen YC, Wang JK, et al. Measuring knowledge of patients with congenital heart disease and their parents: validity of the 'Leuven Knowledge Questionnaire for Congenital Heart Disease'. *Eur J Cardiovasc Nurs* 2012;**11**(1):77-84. doi: 10.1177/1474515111429662

30. Goossens E, Luyckx K, Mommen N, et al. Health risk behaviors in adolescents and emerging adults with congenital heart disease: psychometric properties of the Health Behavior Scale-Congenital Heart Disease. Eur J Cardiovasc Nurs 2013;**12**(6):544-57.

31. Varni JW, Burwinkle TM, Seid M, et al. The PedsQL™* 4.0 as a Pediatric Population Health Measure: Feasibility, Reliability, and Validity. Ambul Pediatr 2003;**3**(6):329-41.

32. Petersen S, Hagglof B, Stenlund H, et al. Psychometric properties of the Swedish PedsQL, Pediatric Quality of Life Inventory 4.0 generic core scales. Acta Paediatr 2009;**98**(9):1504-12.

33. Bratt E-L, Luyckx K, Goossens E, et al. Patient-Reported Health in Young People With Congenital Heart Disease Transitioning to Adulthood. J Adolesc Health 2015;**57**(6):658-65.

34. Sand P, Kljajić M, Sunnegårdh J. The reliability of the Pediatric Quality of Life Inventory 3.0 Cardiac Module™ for Swedish children with congenital heart defects. Nordic Psychology 2013;**65**(3):210-23.

35. Dyer MT, Goldsmith KA, Sharples LS, et al. A review of health utilities using the EQ-5D in studies of cardiovascular disease. Health and Quality of Life Outcomes 2010;**8**(1):1-12.

36. Moons P, Van Deyk K, De Bleser L, et al. Quality of life and health status in adults with congenital heart disease: a direct comparison with healthy counterparts. Eur J Cardiovasc Prev Rehabil 2006;**13**(3):407-13.

For peer review only - http://bmjopen.bmj.com/site/about/guidelines.xhtml

37. Luyckx K, Missotten L, Goossens E, et al. Individual and contextual determinants of quality of life in adolescents with congenital heart disease. *J Adolesc Health* 2012;**51**(2):122-8. doi: 10.1016/j.jadohealth.2011.11.007

38. Apers S, Moons P, Goossens E, et al. Sense of coherence and perceived physical health explain the better quality of life in adolescents with congenital heart disease. *Eur J Cardiovasc Nurs* 2013;**12**(5):475-83. doi: 10.1177/1474515113477955

39. EuroQoL Group. EuroQol--a new facility for the measurement of health-related quality of life. Health Policy 1990;**16**(3):199-208.

40. Moons P, Marquet K, Budts W, et al. Validity, reliability and responsiveness of the "Schedule for the Evaluation of Individual Quality of Life – Direct Weighting" (SEIQoL-DW) in congenital heart disease. Health and Quality of Life Outcomes 2004;**2**(1):1-8.

41. Apers S, Kovacs AH, Luyckx K, et al. Assessment of Patterns of Patient-Reported Outcomes in Adults with Congenital Heart disease - International Study (APPROACH-IS): rationale, design, and methods. Int J Cardiol 2015;**179**:334-42.

42. Hilderson D, Moons P, Van der Elst K, et al. The clinical impact of a brief transition programme for young people with juvenile idiopathic arthritis: results of the DON'T RETARD project. Rheumatology (Oxford) 2016;**55**(1):133-42.

43. World Medical Association. World medical association declaration of helsinki: Ethical principles for medical research involving human subjects. JAMA 2013;**310**(20):2191-94.

44. Moher D, Hopewell S, Schulz KF, et al. CONSORT 2010 explanation and elaboration: updated guidelines for reporting parallel group randomised trials. BMJ 2010;**340**:c869.

45. Bourbonniere MC, Russell DJ, Goldsmith CH. Authorship issues: one research center's experience with developing author guidelines. Am J Occup Ther 2006;**60**(1):111-7.

46. Whellan DJ, Ellis SJ, Kraus WE, et al. Method for establishing authorship in a multicenter clinical trial. Ann Intern Med 2009;**151**(6):414-20.

47. Asp A, Bratt EL, Bramhagen AC. Transfer to Adult Care--Experiences of Young Adults with Congenital Heart Disease. J Pediatr Nurs 2015;**30**(5):e3-10.

For peer review only

48. Burstrom A, Ojmyr-Joelsson M, Bratt EL, et al. Adolescents With Congenital Heart Disease and Their Parents: Needs Before Transfer to Adult Care. J Cardiovasc Nurs 2015.

49. Craig P, Dieppe P, Macintyre S, et al. Developing and evaluating complex interventions: the new Medical Research Council guidance. BMJ : British Medical Journal 2008;**337**:a1655.

50. Erlen JA, Tamres LK, Reynolds N, et al. Assessing usual care in clinical trials. West J Nurs Res 2015;**37**(3):288-98.

51. Sable C, Foster E, Uzark K, et al. Best practices in managing transition to adulthood for adolescents with congenital heart disease: the transition process and medical and psychosocial issues: a scientific statement from the American Heart Association. Circulation 2011;**123**(13):1454-85

For peer review only

Figure legends

Figure 1: Overview of the study design and implementation steps

For peer review only

For peer review only - http://bmjopen.bmj.com/site/about/guidelines.xhtml

Figure 1: Overview of study design and implementation steps

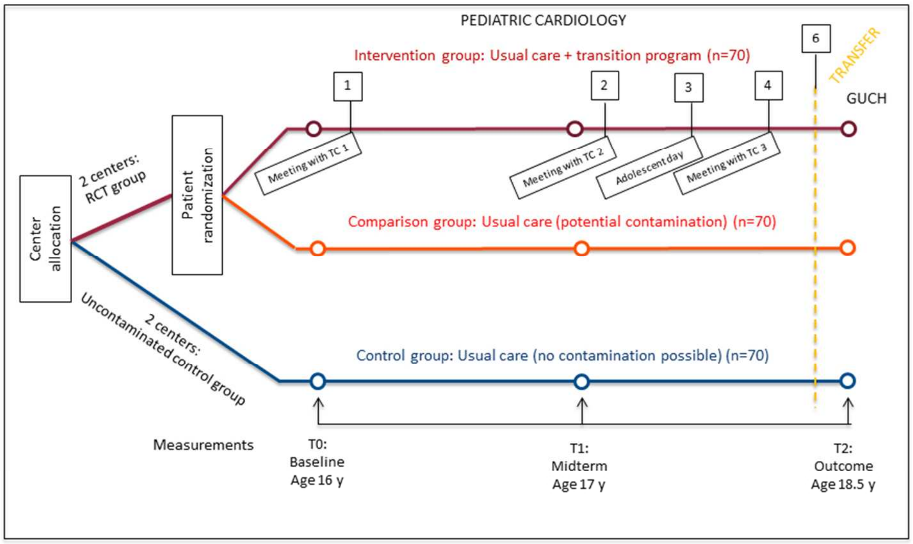

RCT: randomized controlled trial; TC: transition coordinator; GUCH: grown-up congenital heart disease clinic

81x60mm (300 x 300 DPI)

For peer review only - http://bmjopen.bmj.com/site/about/guidelines.xhtml

SPIRIT
STANDARD PROTOCOL ITEMS: RECOMMENDATIONS FOR INTERVENTIONAL TRIALS

SPIRIT 2013 Checklist: Recommended items to address in a clinical trial protocol and related documents*

Section/item	Item No	Description	Addressed on page number
**Administrative information**			
Title	1	Descriptive title identifying the study design, population, interventions, and, if applicable, trial acronym	1
Trial registration	2a	Trial identifier and registry name. If not yet registered, name of intended registry	3
	2b	All items from the World Health Organization Trial Registration Data Set	NA
Protocol version	3	Date and version identifier	NA
Funding	4	Sources and types of financial, material, and other support	28
Roles and responsibilities	5a	Names, affiliations, and roles of protocol contributors	29
	5b	Name and contact information for the trial sponsor	NA
	5c	Role of study sponsor and funders, if any, in study design; collection, management, analysis, and interpretation of data; writing of the report; and the decision to submit the report for publication, including whether they will have ultimate authority over any of these activities	NA
	5d	Composition, roles, and responsibilities of the coordinating centre, steering committee, endpoint adjudication committee, data management team, and other individuals or groups overseeing the trial, if applicable (see Item 21a for data monitoring committee)	NA

For peer review only

For peer review only - http://bmjopen.bmj.com/site/about/guidelines.xhtml

Introduction

Background and rationale	6a	Description of research question and justification for undertaking the trial, including summary of relevant studies (published and unpublished) examining benefits and harms for each intervention	4-5
	6b	Explanation for choice of comparators	4-5
Objectives	7	Specific objectives or hypotheses	5-6
Trial design	8	Description of trial design including type of trial (eg, parallel group, crossover, factorial, single group), allocation ratio, and framework (eg, superiority, equivalence, noninferiority, exploratory)	6

Methods: Participants, interventions, and outcomes

Study setting	9	Description of study settings (eg, community clinic, academic hospital) and list of countries where data will be collected. Reference to where list of study sites can be obtained	6
Eligibility criteria	10	Inclusion and exclusion criteria for participants. If applicable, eligibility criteria for study centres and individuals who will perform the interventions (eg, surgeons, psychotherapists)	7
Interventions	11a	Interventions for each group with sufficient detail to allow replication, including how and when they will be administered	8-10
	11b	Criteria for discontinuing or modifying allocated interventions for a given trial participant (eg, drug dose change in response to harms, participant request, or improving/worsening disease)	NA
	11c	Strategies to improve adherence to intervention protocols, and any procedures for monitoring adherence (eg, drug tablet return, laboratory tests)	11
	11d	Relevant concomitant care and interventions that are permitted or prohibited during the trial	9-10
Outcomes	12	Primary, secondary, and other outcomes, including the specific measurement variable (eg, systolic blood pressure), analysis metric (eg, change from baseline, final value, time to event), method of aggregation (eg, median, proportion), and time point for each outcome. Explanation of the clinical relevance of chosen efficacy and harm outcomes is strongly recommended	13-22
Participant timeline	13	Time schedule of enrolment, interventions (including any run-ins and washouts), assessments, and visits for participants. A schematic diagram is highly recommended (see Figure)	12, (see Figure 1)

For peer review only - http://bmjopen.bmj.com/site/about/guidelines.xhtml

Sample size	14	Estimated number of participants needed to achieve study objectives and how it was determined, including clinical and statistical assumptions supporting any sample size calculations	6-7
Recruitment	15	Strategies for achieving adequate participant enrolment to reach target sample size	12
**Methods: Assignment of interventions (for controlled trials)**			
Allocation:			
Sequence generation	16a	Method of generating the allocation sequence (eg, computer-generated random numbers), and list of any factors for stratification. To reduce predictability of a random sequence, details of any planned restriction (eg, blocking) should be provided in a separate document that is unavailable to those who enrol participants or assign interventions	7
Allocation concealment mechanism	16b	Mechanism of implementing the allocation sequence (eg, central telephone; sequentially numbered, opaque, sealed envelopes), describing any steps to conceal the sequence until interventions are assigned	7
Implementation	16c	Who will generate the allocation sequence, who will enrol participants, and who will assign participants to interventions	Randomization section: 7-8
Blinding (masking)	17a	Who will be blinded after assignment to interventions (eg, trial participants, care providers, outcome assessors, data analysts), and how	10
	17b	If blinded, circumstances under which unblinding is permissible, and procedure for revealing a participant's allocated intervention during the trial	NA
**Methods: Data collection, management, and analysis**			
Data collection methods	18a	Plans for assessment and collection of outcome, baseline, and other trial data, including any related processes to promote data quality (eg, duplicate measurements, training of assessors) and a description of study instruments (eg, questionnaires, laboratory tests) along with their reliability and validity, if known. Reference to where data collection forms can be found, if not in the protocol	12, 14-18
	18b	Plans to promote participant retention and complete follow-up, including list of any outcome data to be collected for participants who discontinue or deviate from intervention protocols	12

For peer review only - http://bmjopen.bmj.com/site/about/guidelines.xhtml

Data management	19	Plans for data entry, coding, security, and storage, including any related processes to promote data quality (eg, double data entry; range checks for data values). Reference to where details of data management procedures can be found, if not in the protocol	22
Statistical methods	20a	Statistical methods for analysing primary and secondary outcomes. Reference to where other details of the statistical analysis plan can be found, if not in the protocol	22-23
	20b	Methods for any additional analyses (eg, subgroup and adjusted analyses)	22-23
	20c	Definition of analysis population relating to protocol non-adherence (eg, as randomised analysis), and any statistical methods to handle missing data (eg, multiple imputation)	NR
**Methods: Monitoring**			
Data monitoring	21a	Composition of data monitoring committee (DMC); summary of its role and reporting structure; statement of whether it is independent from the sponsor and competing interests; and reference to where further details about its charter can be found, if not in the protocol. Alternatively, an explanation of why a DMC is not needed	NA
	21b	Description of any interim analyses and stopping guidelines, including who will have access to these interim results and make the final decision to terminate the trial	NA
Harms	22	Plans for collecting, assessing, reporting, and managing solicited and spontaneously reported adverse events and other unintended effects of trial interventions or trial conduct	24
Auditing	23	Frequency and procedures for auditing trial conduct, if any, and whether the process will be independent from investigators and the sponsor	NA
**Ethics and dissemination**			
Research ethics approval	24	Plans for seeking research ethics committee/institutional review board (REC/IRB) approval	23
Protocol amendments	25	Plans for communicating important protocol modifications (eg, changes to eligibility criteria, outcomes, analyses) to relevant parties (eg, investigators, REC/IRBs, trial participants, trial registries, journals, regulators)	NA

For peer review only - http://bmjopen.bmj.com/site/about/guidelines.xhtml

Consent or assent	26a	Who will obtain informed consent or assent from potential trial participants or authorised surrogates, and how (see Item 32)	21
	26b	Additional consent provisions for collection and use of participant data and biological specimens in ancillary studies, if applicable	NA
Confidentiality	27	How personal information about potential and enrolled participants will be collected, shared, and maintained in order to protect confidentiality before, during, and after the trial	23
Declaration of interests	28	Financial and other competing interests for principal investigators for the overall trial and each study site	28
Access to data	29	Statement of who will have access to the final trial dataset, and disclosure of contractual agreements that limit such access for investigators	22
Ancillary and post-trial care	30	Provisions, if any, for ancillary and post-trial care, and for compensation to those who suffer harm from trial participation	NA
Dissemination policy	31a	Plans for investigators and sponsor to communicate trial results to participants, healthcare professionals, the public, and other relevant groups (eg, via publication, reporting in results databases, or other data sharing arrangements), including any publication restrictions	24
	31b	Authorship eligibility guidelines and any intended use of professional writers	24
	31c	Plans, if any, for granting public access to the full protocol, participant-level dataset, and statistical code	NA
**Appendices**			
Informed consent materials	32	Model consent form and other related documentation given to participants and authorised surrogates	Available upon request
Biological specimens	33	Plans for collection, laboratory evaluation, and storage of biological specimens for genetic or molecular analysis in the current trial and for future use in ancillary studies, if applicable	NA

*It is strongly recommended that this checklist be read in conjunction with the SPIRIT 2013 Explanation & Elaboration for important clarification on the items. Amendments to the protocol should be tracked and dated. The SPIRIT checklist is copyrighted by the SPIRIT Group under the Creative Commons "Attribution-NonCommercial-NoDerivs 3.0 Unported" license.

For peer review only - http://bmjopen.bmj.com/site/about/guidelines.xhtml
